# Integration of molecules and new fossils supports a Triassic origin for Lepidosauria (lizards, snakes, and tuatara)

**DOI:** 10.1186/1471-2148-13-208

**Published:** 2013-09-25

**Authors:** Marc EH Jones, Cajsa Lisa Anderson, Christy A Hipsley, Johannes Müller, Susan E Evans, Rainer R Schoch

**Affiliations:** 1 Research Department of Cell and Developmental Biology, Anatomy Building, UCL, University College London, Gower Street, London WCIE 6BT, UK; 2 School of Earth and Environmental Sciences, The University of Adelaide, North Terrace, Adelaide, South Australia 5005, Australia; 3 University of Gothenburg, Department of Plant and Environmental Sciences, Gothenburg, Sweden; 4 Museum für Naturkunde – Leibniz-Institut für Evolutions- und Biodiversitätsforschung an der Humboldt-Universität zu Berlin, Berlin, Germany; 5 Staatliches Museum für Naturkunde, Rosenstein 1, D-70191, Stuttgart, Germany; 6 Berlin-Brandenburg Institute of Advanced Biodiversity Research (BBIB), 14195 Berlin, Germany

**Keywords:** Dating, Fossil, Jurassic, Lepidosauria, Lizards, Molecular, Origin, Squamata, Triassic, Tuatara

## Abstract

**Background:**

Lepidosauria (lizards, snakes, tuatara) is a globally distributed and ecologically important group of over 9,000 reptile species. The earliest fossil records are currently restricted to the Late Triassic and often dated to 227 million years ago (Mya). As these early records include taxa that are relatively derived in their morphology (e.g. *Brachyrhinodon*), an earlier unknown history of Lepidosauria is implied. However, molecular age estimates for Lepidosauria have been problematic; dates for the most recent common ancestor of all lepidosaurs range between approximately 226 and 289 Mya whereas estimates for crown-group Squamata (lizards and snakes) vary more dramatically: 179 to 294 Mya. This uncertainty restricts inferences regarding the patterns of diversification and evolution of Lepidosauria as a whole.

**Results:**

Here we report on a rhynchocephalian fossil from the Middle Triassic of Germany (Vellberg) that represents the oldest known record of a lepidosaur from anywhere in the world. Reliably dated to 238–240 Mya, this material is about 12 million years older than previously known lepidosaur records and is older than some but not all molecular clock estimates for the origin of lepidosaurs. Using RAG1 sequence data from 76 extant taxa and the new fossil specimens two of several calibrations, we estimate that the most recent common ancestor of Lepidosauria lived at least 242 Mya (238–249.5), and crown-group Squamata originated around 193 Mya (176–213).

**Conclusion:**

A Early/Middle Triassic date for the origin of Lepidosauria disagrees with previous estimates deep within the Permian and suggests the group evolved as part of the faunal recovery after the end-Permain mass extinction as the climate became more humid. Our origin time for crown-group Squamata coincides with shifts towards warmer climates and dramatic changes in fauna and flora. Most major subclades within Squamata originated in the Cretaceous postdating major continental fragmentation. The Vellberg fossil locality is expected to become an important resource for providing a more balanced picture of the Triassic and for bridging gaps in the fossil record of several other major vertebrate groups.

## Background

Lepidosauria (lizards, snake, tuatara) currently have a global distribution, encompass >9000 species, and fill a variety of ecological niches [1,2]. The vast majority of this diversity comprises lizards and snakes (Squamata). By contrast, their sister group, Rhynchocephalia, is represented by a single extant species, *Sphenodon punctatus*, the New Zealand tuatara [3,4]. The fossil record suggests for the first half of the Mesozoic, Rhynchocephalia was the more successful lepidosaur group but the earliest history of Lepidosauria remains incompletely known [5-10]. An accurate estimate for when this clade originated is crucial for appreciating the ecological context in which it first evolved in addition to its subsequent diversification. Currently, the oldest fossil records of Lepidosauria are rhynchocephalian and Late Triassic in age (228–235 Mya, Carnian): *Brachyrhinodon* from the Lossiemouth Sandstone Formation of Scotland, UK [11], and a partial jaws from the Vinita Formation (previously the 'Turkey Branch’), Virginia, USA ([12,13], specimen figured in [14]) that include material reported to resemble *Diphydontosaurus* from the Late Triassic of England [12,15]. Now that *Tikiguania*[16] is considered to be modern rather than Late Triassic in age [17] the earliest putative squamate fossils are from the Early Jurassic of India [18]. However, as rhynchocephalians were present in the Late Triassic, stem lineage representatives of their sister taxon Squamata must also have been present concurrently [9].

Problematically, the earliest known lepidosaurs are already derived in several aspects of their anatomy [9]. Cladistic analyses consistently nest *Brachyrhinodon* amongst derived rhynchocephalians [19-21]. *Diphydontosaurus* is one of the least phylogenetically nested rhynchocephalians, but the stout teeth with prominent radial ridges of the Vinita specimen [14] suggest a closer affinity to the more derived *Planocephalosaurus* from the Late Triassic of the UK [22]. Also other slightly younger Late Triassic Rhynchocephalia are both widespread and diverse [5,10,23-25]. Hence, the success of Late Triassic Rhynchocephalia suggests either a rapid diversification of the clade or alternatively an older unknown history during the Early and Middle Triassic [24-26]. Unfortunately, this crucial interval remains cryptic due to the rarity of fossil deposits of the correct age and with suitable preservational potential for small vertebrates [5,9].

Until recently, the record of stem-lepidosaurs was not very helpful to the question of lepidosaur origins [5]. *Paliguana* from the Early Triassic of South Africa is from the appropriate time interval but the specimen is badly damaged and provides little data [9,27]. The aquatic *Marmoretta* (Middle Jurassic of the UK, [26,28]), parachuting/gliding kuehneosaurs (Late Triassic of USA and the UK, [29,30]), and burrowing *Tamaulipasaurus* (Early Jurassic of Mexico, [31]) are all younger than or coeval with the oldest lepidosaurs. The Middle Triassic *Megachirella*[32] is older but of questionable affinity [5]. The newly described kuehneosaur *Pamelina*[33] and the less specialised *Sophineta*[34] from the Early Triassic of Poland confirm that stem-lepidosaurs were present and had diversified by at least the Early Triassic.

Aside from fossils, molecular dating provides a complimentary means of estimating the origin of Lepidosauria. Initial calculations by Kumar and Hedges [35] based on amino acid sequences provided a broad estimate of 276±54.4 Mya located deep within the Permian (Table 1, Additional file 1). Several subsequent analyses using more recent methods have also recovered estimates from within the Permian, 289 and 265 Mya [36-39]. However, other molecular dating analyses provide dates in the Late or Middle Triassic with one as recent/shallow as 226 Mya [39-43]. This range of estimates is far more disparate than those based on the fossil record and morphological characters which suggest an Early to Middle Triassic origin time (e.g. [9,26]). Although the lizard-tuatara node was not listed as a potential calibration for the animal tree of life by Benton & Donoghue [44], it was by Benton [45], and some analyses have used the earliest currently known lepidosaur fossils to constrain divergence times for investigating the origins of both squamates and amniotes [37,38,40-43]. Despite uncertainty regarding the exact age of the Lossiemouth Sandstone Formation and the likely older Vinita Formation (e.g. [11,13]), the date of 227 or 228 Mya is often used (e.g. [41,42]) or suggested [45]. One recent analysis [43] used 222.8 Mya based on dates for the Upper-Carnian boundary found in Gradstein et al. [46]. However, revised stratigraphic work suggests the age of this boundary is older [47].

**Table 1:** Summary of previous molecular divergence estimates

**Analysis**	**Material**	**No. of squamate taxa**	**Dating software and/or method**	**Age crown Lepidosauria**	**Age crown Squamata**
Albert et al. [38]	mtDNA (13 genes)	27	r8s, Penalized Likelihood	289±5	281
Albert et al. [38]	mtDNA (13 genes)	27	“Multidivtime”, Bayesian autocorrelated clock	272±20	259
Alfaro et al. [3]	nDNA: RAG-1	35	BEAST, Bayesian uncorrelated lognormal clock	246 (208-275)	mid TR - mid JU
Gorr et al. [40]	α haemoglobin chains	3 | 6	Strict clock (least-squares regression)	233	n/a
Gorr et al. [40]	β haemoglobin chains	9	Strict clock (least-squares regression)	226	~194
Hipsley et al. [42]	mtDNA and nDNA (5 genes)	40 ^1^	TreeTime, Bayesian uncorrelated lognormal clock	238±10	n/a
Hugall et al. [36]	nDNA: RAG-1	36	r8s, Penalized Likelihood	250-268±12 ^2^	171-190* ±14
Hugall et al. [36]	nDNA: RAG-1, translated	36	r8s, Penalized Likelihood	261-275±17 ^2^	184-201* ±19
Janke et al. [48]	mtDNA	2	Strict clock (after pruning of taxa)	n/a	294 ^3^
Kumar and Hedges [35]	Amino acid sequences (5 genes)	?	Strict clock (after pruning of heterogeneous sequences)	276±54.4	n/a
Kumazawa [37]	mtDNA	24	“Multidivtime”, Bayesian autocorrelated clock	~260-290	~215-255
Mulcahy et al. [43]	mtDNA and nDNA (RAG-1)	64	BEAST, Bayesian uncorrelated lognormal clock	~233 (223-243)	180 (160-198)
Mulcahy et al. [43]	mtDNA and nDNA (RAG-1)	64	r8s, Penalized Likelihood	~275 (na)	191.8 (186-194)
Okajima & Kumazawa [49]	mtDNA	22	“Multidivtime”, Bayesian autocorrelated clock	n/a	240 (220-260)
Pyron [39]	nDNA: RAG-1 ^4^	44	BEAST, Bayesian uncorrelated lognormal clock	236 (212-253)	189 (163-213)
Pyron [39]	nDNA: RAG-1 ^5^	44	BEAST, Bayesian uncorrelated lognormal clock	265 (240-290)	208 (179-234)
Shen et al. [50]	mtDNA and nDNA	5	“Multidivtime”, Bayesian autocorrelated clock	n/a	205 (180-228)
Vidal and Hedges [51]	nDNA: C-mos, RAG-1	19	“Multidivtime”, Bayesian autocorrelated clock	< 251	240 (221-251)
Wiens et al. [41]	nDNA: RAG-1 ^6^	261 ^7^	r8s, Penalized Likelihood	227 ^8^	179 ±5.5

Annotations: ^1^ focused on lacertids, ^2^ range of four different estimates provided by varying the number of calibration points, ^3^*Sphenodon* was not included amongst the taxa therefore the estimate better corresponds to one for Lepidosauromorpha, ^4^ four fossil calibrations from Müller and Reisz [52], ^5^ five fossil calibrations from Hugall et al. [36], ^6^ supertree approach, ^7^ focused on taxa with a snake-like bodyform, ^8^ used as fixed calibration point. Abbreviations: *JU* Jurassic, *TR* Triassic.

The origin time of crown-group Squamata (all living squamates and their most recent common ancestor) has received an even greater degree of attention [3,36-39,41,43,48-51,53]. Squamates are an ecologically important component of our modern fauna but the timing and thus ecological context of their initial evolution remains poorly understood [5,6,9]. Current estimates for crown-group squamate origins vary by 120 million years (Table 1) with the oldest/deepest date being 294 Mya [48] and most recent/shallow being 179 Mya [41]. This represents a 60% difference between these two points in time (Early Permian or Early Jurassic), when many aspects of the biosphere were radically different: continental distributions, palaeoclimates, vegetation, macrofaunas, and potential prey and predator species (e.g. [47,54-62]). Moreover, these two estimates straddle the end Permian and (less well understood) end Triassic mass-extinction events, both of which significantly impacted terrestrial vertebrate communities (e.g. [47,54,57,63-68]).

Constraining the origination times of Lepidosauria and crown-group Squamata is also important for evaluating divergences within Squamata and for improving the accuracy of molecular dating analyses for the group as a whole (e.g. [39,43]). It has been shown that the most important factor for improving molecular age estimates is the amount and quality of age constraints (e.g. [69-71]), and studies assessing the relationship between historical events and biological evolution (e.g. clade divergence, adaptive radiations, biogeography, species richness patterns) rely on date constraints being accurate (e.g. [3,42,72-74]). The discovery of any Early or Middle Triassic lepidosaur fossil material would clearly have implications for the ages of early lepidosaur divergences and associated evolutionary history.

Here we report a new rhynchocephalian from the Middle Triassic of Germany (240 Mya) that predates previously known lepidosaur material by about 12 million years. We describe the two partial dentaries in detail and include them in a cladistic analysis based on both old and new morphological characters to test their lepidosaur affinities. We also carry out a molecular divergence analysis using the new fossil and 13 other reliable amniote fossils, to provide a new framework for divergence times for Lepidosauria, Squamata, and subgroups within the latter.

### Institutional abbreviations

SMNS, Staatliches Museum für Naturkunde, Stuttgart, Baden-Württemberg, Germany.

## Methods

The new fossil material described here comprises two partial dentaries: a right bone exposed in lateral view bearing six teeth (SMNS 91060) and a left bone exposed in lingual view bearing two large teeth posteriorly and at least seven distinctly smaller teeth anteriorly (SMNS 91061).

### Geographic and stratigraphic provenance

**Figure 1:**
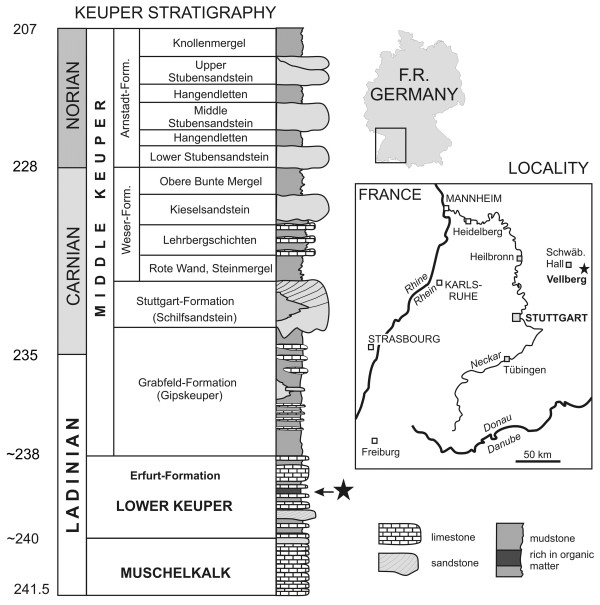
**Geographic and stratigraphic data for the Vellberg jaws.** The lepidosaur bearing horizon in the Lower Keuper is marked with a star.

Both specimens were found in the same 50–100 mm thick mudstone layer at the top of the Untere Graue Mergel (lower grey marls) of the Lower Keuper (Erfurt Formation) (Figure 1). This corresponds to layer 6 of Schoch [75] which is known only from the Vellberg locality, southern Germany. Cyclostratigraphic data [76] suggests the Erfurt Fm is between 239 and 240 Mya which corresponds to the Ladinian part of the Middle Triassic [47,77]. Kozur and Bachman [78] suggest a slightly earlier date of 238–238.8 Mya for this unit based on zircon U-Pb dating.

The locality preserves deposits from a freshwater lake a few kilometers in diameter. Contemporaneous exposures in the vicinity lack layer 6 and show evidence for large brackish swamps instead. The local fauna was diverse and included actinopterygians, lungfishes, coelacanths, temnospondyls, sauropterygians, and archosaurs of various sizes [75,79-88]. Local climate was probably monsoonal including both dry and humid intervals [89,90].

### Morphological examination

Specimens were examined using a Wild stereobinocular microscope and drawn using a *camera lucida* attachment. Specimen SMNS 91060 was also examined using a JEOL JSM-5410LV Scanning Electron Microscope in the Research Department of Cell and Developmental Biology at University College London. Both specimens were scanned using a X-Tek HMX 160 micro CT scanner in the Department of Engineering at the University of Hull using the following parameters: scan energy 80kV, uA 22 (SMNS 91060) and uA 20 (SMNS 91061), aperture 75%, 1000 projections averaging 16 frames per projection. To reduce beam hardening the x-rays were filtered through a 0.1 mm copper plate. Voxel resolution was 0.0227 mm^3^ for SMNS 91060 and 0.0374 mm^3^ for SMNS 91061. The CT models (Additional files 2, 3 and Additional file 1: Figure S1.1) were constructed using the software Amira 4.1 (Mercury Computer Systems Inc, USA).

### Phylogenetic placement of the Vellberg fossils

Twenty-two taxa were used for phylogenetic assessment of the Vellberg jaws. Of these, 20 are fossil taxa, 15 represent ingroup taxa and 7 outgroup taxa (Additional file 1). Squamata was used as a metataxon because the early fossil record of this group remains poor. Modern examplar taxa were not used to represent Squamata, because within this diverse group it is uncertain what the plesiomorphic states are and which taxa would best represent the group as a whole.

The 22 taxa were coded using 100 characters. Many of the characters have a long history of usage in cladistic studies and date back to work by Evans [91,92], Whiteside [15], Benton [93] and Gauthier *et al*. [94]. Others characters include those that were added and modified during a number of subsequent studies (e.g. [11,19-21]). Characters 1 to 77 (see Additional file 1) broadly correspond to those used by Evans [33] and Evans and Borsuk-Bialynicka [34]. These were in error said to be listed in Waldman and Evans [28] but in actuality refer to part of the analysis that was removed prior to publication.

Despite the number of characters used in previous analyses, this matrix should be treated as new because several characters have been modified to accommodate both rhynchocephalians and stem group lepidosauromorphs.

The data matrix was analyzed using PAUP* 4.0b10 [95] and MrBayes [96]. All characters were equally weighted and unordered. In the few cases where taxa exhibited multiple states for the same character, the state was treated as uncertain (by default, PAUP* treats uncertain multistate characters as polymorphism, whilst MrBayes treats them as total uncertainty, which could potentially lead to larger differences in inferred trees if the matrix contains many multiple state characters). *Petrolacosaurus* was used as the outgroup. Bootstrap support for clades found by PAUP* were calculated from 1000 replicates of heuristic search using TBR and random addition. MrBayes was run for 1 million generations with sample frequency 1000, 3 runs with 4 chains each, and the majority rule consensus tree was calculated after a 50% burnin. For characters and matrix, see Additional file 1. The matrix is also deposited in the Dryad data repository (http://datadryad.org/), with the Digital Object Identifier (DOI) of http://dx.doi.org/10.5061/dryad.gr573

### Molecular divergence dating

We compiled a dataset of RAG1 nuclear gene sequences from GenBank for 76 extant amniote taxa (Additional file 4). This comprised *Sphenodon punctatus* (Rhynchocephalia), 62 lizards and snakes (Squamata), four Testudines, four Aves and three Crocodylia (see Additional file 4). Two mammals (one marsupial and one monotreme) served as outgroups. Sequences were aligned using the ClustalW option in SeaView [97].

For choosing the molecular substitution model we analysed the data using MrModelTest v2 [98], and based on the Akaike Information Criterion, the most parameter-rich model GTR+G+I was suggested. However, we chose the less complex model GTR+G, because althoughGTR+G+I would improve the model’s fit to the data it also seems to cause convergence difficulties rather than improving the phylogenetic reconstruction and dating. Several studies have shown that the gamma shape parameter and the invariant sites parameter are highly correlated and even considered to be “pathological” when estimated together [99,100]. The combination of G+I can overestimate the rate of molecular evolution and, consequently, affect the estimation of divergence times.

For phylogenetic reconstruction and divergence time estimation, the BEAST [101] software package (version 1.7.3) was used. The methods implemented in BEAST make it possible to infer tree topology simultaneously with ages. However, as our data set contains a large number of fossil constraints as well as long branches / heterogeneous rates across the phylogeny, the initial UPGMA starting tree inferred by BEAST did not fit the data, causing the initial likelihood to be zero. This problem is solved by providing a starting tree that is fully bifurcating and not in conflict with the data and prior assumptions.

To obtain a starting tree we ran a MrBayes analysis [96] under the GTR+G model, three runs and three chains over five million generations. After discarding a burn in of 50% we filtered the output trees using PAUP* and a set of “soft” backbone constraints (polytomies representing uncertain parts of the topology), so that all trees were consistent with current knowledge of reptile phylogeny and that subtrees that need to be monophyletic for the calibration points were not violated. (For the unfiltered majority rule consensus tree with posterior probabilities, see Additional file 5.) One random tree from this set of filtered trees was used for dating using the penalized likelihood method (PL) implemented in the r8s software [102]. To obtain a starting tree for BEAST it was further necessary to heavily constrain the nodes in the PL analyses, and 6 fossils were used as both minimum (the fossil age) and maximum (the fossil age plus 20%) ages.

For the final BEAST analysis the uncorrelated lognormally distributed clock model was used [103], with the Yule birth rate as the general tree prior.

In total 14 fossils were used to specify informative priors on internal node divergence times. These were chosen following the recommendations on fossil calibrations of Parham et al. [74]. Calibrated nodes are: (CNX) Archosauromorpha–Lepidosauromorpha, 255 Mya, based on *Protorosaurus* sp., the oldest known archosauromorph [104]; (CNY) *Alligator*–*Passer montanus*, 247 Mya (to 256 Mya), based on oldest known certain archosaur *Ctenosauriscus koeneni*[105]; (CN1) *Sphenodon*–*Varanus* (origin of Lepidosauria, the tuatata-lizard split) 238 Mya, based on the new fossil jaws described here; (CN2) *Eublepharis*–*Sphaerodactylus* (origin of Gekkonidae), 44 Mya, based on *Yantarogekko balticus*, the earliest certain gekkonid [106,107]; (CN3) *Xantusia*–*Cordylus*, 61 Mya, based on *Palaeoxantusia fera*, the earliest known xantusiid [47,108-110]; (CN4) Lacertidae–Amphisbaenia, 61 Mya, based on *Plesiorhineura tsentasi*, the earliest certain amphisbaenian [108-111]; (CN5) *Python*–*Elgaria* (Serpentes-Anguimorpha), 148 Mya, based on *Dorsetisaurus* sp., the earliest known anguimorph [112-114]; (CN6) *Varanus*–*Lanthanotus*, 48 Mya, based on *Saniwa ensidens* an immediate sister taxon to *Varanus*[114-117]; (CN7) *Heloderma*–*Anniella*, 98 Mya, based on *Primaderma nessovi* which represents the oldest fossil taxon more closely related to *Heloderma* than to any other living taxon [114,118,119]; (CN8) *Elgaria*–*Ophisaurus*, 33 Mya, based on fossil material referable to *Ophisaurus* sp. from the UK [47,120,121]; (CN9) *Chamaeleo*–*Calumma*, 19 Mya, based on fossil material referable to *Chamaeleo* sp. from the Czech Republic [122,123]; (CN10) *Physignathus*–*Ctenophorus*, 16 Mya, based on material referable to *Physignathus* sp. from Australia [124-126]; (CN11) *Gambelia*–*Anolis*, 48 Mya, based on *Afairiguana avius* the oldest pleurodontan iguanian [117,118,127,128]; and (CN12) *Shinisaurus*–*Elgaria*, 128 Mya, based on *Dalinghosaurus longidigitus* which may be more closely related to *Shinisaurus* than to any other living squamate [114,129,130]. For the full justification of each of the fossil specimens and their age see Additional file 1. We also ran three different schemes of fossil-based cross validations [131] on the 14 fossils used in the dating.

All fossils were used as a hard minimum age constraint to the node below the hypothesized branching of the fossil lineage. For the prior distributions of ages the exponential prior was used and the mean set consistently to 4.0 for all constraints (Table 2). In absolute ages this prior distribution means an age interval of about 15–20 million years, with low probability of being older. Monophyly of groups constrained by fossils was enforced.

**Table 2:** Summary of the prior and posterior ages for the constrained nodes

**Constrained nodes**	**Split**	**Minimum age of fossil constraint**	**Median posterior (calculated age)**	**95% HPD lower**	**95% HPD upper**
X	Archosauromorpha*–*Lepidosauromorpha	255	271	259	285.2
Y	Crown Archosauria *sensu stricto*	247	248.3	247	252.8
1	*Sphenodon–Varanus* (Lepidosauria)	238	240.8	238	249.6
2	*Eublepharis–Sphaerodactylus* (Gekkonidae)	44	50.5	44	63.3
3	*Xantusia–Cordylus*	61	67	61	84.3
4	Lacertidae*–*Amphisbaenia	61	66.1	61	80.9
5	*Python–Elgaria* (Anguimorpha)	148	150.3	148	156.8
6	*Varanu*s*–Lanthanotus*	48	50.5	48	58.3
7	*Heloderma–Anniella*	98	100.4	98	108
8	*Elgaria–Ophisaurus*	33	35.3	33	42.1
9	*Chamaeleo–Calumma* (chameleons)	19	21.8	19	29.6
10	*Physignatus–Ctenophorus*	16	18.9	16	26.9
11	*Gambelia–Anolis*	48	50.5	48	58
12	*Shinisaurus–Elgaria*	128	129.6	128	134.4

The minimum ages of the fossils were used as hard bounds, and prior ages set as exponentially distributed with a mean = 4.0. The posterior (calculated) ages are listed as median, 95% HDP lower and 95% HDP upper.

Fifty million generations were run and logged every 1000 generations. Convergence and effective sample size (ESS) for parameters were checked with Tracer (version 1.5), with a burn in of 10%. For further confirmation of convergence, the analysis was run several times, with identical settings as well as slightly different values for the operators. Median ages and credibility intervals (CI) were calculated using TreeAnnotator. The XML-file for the BEAST analysis as well as the RAG1 nexus alignment are deposited in the Dryad data repository (http://datadryad.org/), with the DOI of http://dx.doi.org/10.5061/dryad.gr573.

## Results

### Systematic palaeontology

Lepidosauria Haeckel [132]*sensu* Gauthier *et al*. [94].

Rhynchocephalia Günther [133]*sensu* Gauthier *et al*. [94].

cf. *Diphydontosarus* sp.

### Vellberg jaws – description

**Figure 2:**
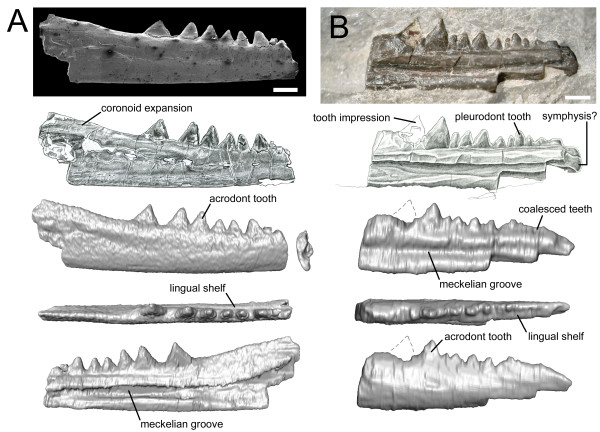
**Partial rhynchocephalian dentaries from the Vellberg locality of Germany. A.** Dentary SMS 91060. From top to bottom: SEM of labial aspect, drawing of labial aspect, CT model in labial, dorsal and lingual view. **B.** Dentary SMS 91061. From top to bottom: photo of lingual aspect, drawing of lingual aspect, CT model in lingual, dorsal and labial view. Scale bars equal 1 mm.

Although incomplete, the dentaries are well preserved. The first specimen (SMNS 91060) bears six laterally compressed teeth that are triangular in lateral profile, sit on the crest of the jaw bone (acrodont implantation), and are fused so that the boundary between tooth and jaw bone is indistinct (Figure 2A; Additional file 2). The remains of a smaller seventh tooth are present anteriorly but it is broken. The dentary extends posteriorly beyond the tooth row and expands dorsally so that the dorsoventral height of the element is twice that of the available anterior end. Six ovoid foramina lie beneath the tooth row along the jaw labially. The Meckelian canal is open and located at a level midway between the tooth row and ventral margin of the jaw.

The second specimen (SMNS 91061) shows evidence of two acrodont teeth: the posteriormost tooth is missing, but the surrounding matrix bears a clear impression of a mediolaterally compressed cone, and an ovoid base is clearly visible in dorsal view (Figure 2B; Additional file 4: Video S4). The two posterior teeth are preceeded by seven teeth which are smaller and more columnar. These latter teeth are less clearly fused to the bone, sit against a low labial wall (weak pleurodont implantation). Anterior to these seven teeth is a short series of small teeth that appear to have coalesced. In rhynchocephalians and some derived squamates with acrodont teeth, new teeth are added to the rear of the jaw with growth (e.g. [23,134]). Therefore, differences in the number of large posterior teeth may relate to ontogeny and both specimens probably represent the same taxon (Additional file 1: Figure S1.2). The anterior end of this second dentary is rounded and may represent part of the symphysial region. If this is correct, it suggests that an adult animal possessed about 14 teeth with an equal number of acrodont and subpleurodont teeth. A facet for a splenial does not appear to be present and the Meckelian canal is similar in position to that of SMNS 91060.

In both specimens, a shelf is present lingual to the base of the tooth row and this diminishes posteriorly. The teeth lack any obvious ornamentation or ridging.

### Comparisons with other taxa

The jaws of stem-lepidosaurs are gracile and bear large numbers of small, weakly implanted acuminate teeth [28,33,34]. In contrast, the Vellberg dentaries demonstrate several features supporting attribution to Lepidosauria and, more particularly, Rhynchocephalia, including possession of a coronoid expansion and a lingual subdental shelf [34]. As in rhynchocephalians with presumed plesiomorphic characters, such as *Gephyrosaurus* from the Early Jurassic of Wales (UK) [91,135], the dentition is regionalised into anterior and posterior series based on tooth size, shape and implantation [15]. The posterior teeth are larger than the anterior teeth, labiolingually compressed and triangular in profile, sit on the crest of the jaw bone (acrodonty), and are fused so that the boundary between tooth and bone is indistinct (Figure 2A, B). The anterior teeth are smaller, more columnar in shape, and sit against a low labial wall (weakly pleurodont) (Figure 2B). Both acrodonty and pleurodonty are derived character states of lepidosaurs [9,34], but only rarely do they occur together: the Vellberg jaws, *Diphydontosaurus*, reportedly *Whitakersaurus* from the Late Triassic of USA [24], and some agamid lizards (e.g. [134]). Two further characters of the dentition support attribution to Rhynchocephalia. The first is the apparent absence or slow pace of tooth replacement, as evidenced by the lack of gaps in the tooth row [34,91,135]. The second is the apparent coalescence of the anteriormost teeth (Figure 2B), a feature reminiscent of rhynchocephalians crownward of *Diphydontosaurus* that lay down additional hard tissues around teeth during life (e.g. [23,136]).

Phylogenetic affinity within Rhynchocephalia is harder to determine. Assuming the anterior end of the dentary is present, the tooth number (about 14) is less than that found in *Gephyrosaurus* (30–40), *Diphydontosaurus* (20–25), *Whitakersaurus* (18-<20), and a juvenile animal from the Late Triassic of Italy referred to *Diphydontosaurus*[15,24,91,137]. Tooth number is more similar to *Planocephalosaurus* (<15), but this taxon has stouter teeth and a characteristically large posterior tooth bearing an incipient flange [22]. The teeth of the Vellberg specimens lack the striations apparent in the Vinita specimen [14] and reported in *Whitakersaurus*, as well as the flanges or obvious wear facet of derived rhynchocephalians such as clevosaurs, eilenodontines, and sphenodontines [10,136,138]. Another character often found in derived rhynchocephalians but absent from the Vellberg jaws is a labial skirt of secondary bone running along the dentary [15,136]. Overall, observations support the attribution of the Vellberg jaws to Rhynchocephalia in a phylogenetic position close to that of *Diphydontosaurus* or the less well known *Whitakersaurus*.

Two other groups of Triassic reptiles possess teeth that are acrodont and strongly fused: trilophosaurs and procolophonids [139,140]. However, the Vellberg jaws differ from those of either group in several ways. The teeth lack the ventral constriction, bulbous nature and slightly raised base often found in trilophosaur and procolophonid teeth [140,141]; they are not transversely expanded or separated by slot-like gaps [140-144]; and the slender elongate build of the Vellberg jaws is also inconsistent with their identification as procolophonid [139]. A procolophonid jaw has been described from Vellberg [84] and in contrast to the lepidosaur specimens this specimen exhibits a steeply rising coronoid process, bulbous teeth with ridged tooth tips, and a mesiodistal base dimension of >2 mm.

### Vellberg jaws – morphology based phylogenetic analysis

**Figure 3:**
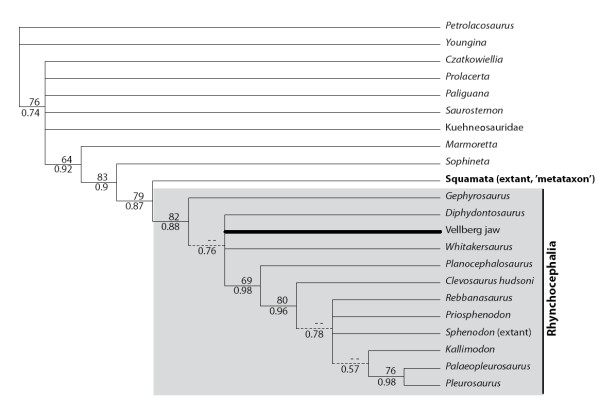
**Phylogenetic relationships of the fossil jaws based on morphological data from living and extinct taxa.** 50% majority rule consensus tree inferred by MrBayes 3.1. Numbers below lines denote posterior probabilities. Numbers above lines denote bootstrap support values obtained from 1000 bootstrap replicates using parsimony criterion in PAUP*. Dashed lines indicate branches found by MrBayes but collapsed in the parsimony analysis, i.e. have less than 50% bootstrap support.

The phylogenetic analysis employing 100 morphological characters and 22 taxa places the Vellberg jaws within Lepidosauria and Rhynchocephalia, confirming that these jaws represent the earliest known lepidosaur material. PAUP* and MrBayes gave essentially the same topology with a small difference in resolution, which was expected as the prior assumptions for morphological characters approximate parsimony. The phylogeny with bootstrap support and posterior probability values, as well as indication of the nodes where the methods give different resolution, is given in Figure 3.

The Bayesian analysis places *Gephyrosaurus* as the sister taxon to the remaining genera in Rhynchocephalia, but with poor support (posterior probability of 0.65). *Diphydontosaurus*, *Whitakersaurus*, and the Vellberg jaw are placed in a polytomy with a well supported monophyletic clade of more derived rhynchocephalians. The lack of resolution at this node is not surprising, as the Vellberg material and *Whitakersaurus* can only be coded for a relatively small number of jaw characters and several of those features represent synapomorphies for Rhynchocephalia as a whole. Within the derived group, *Planocephalosaurus* is well supported as sister taxon to the rest, followed by *Clevosaurus*. The Jurassic pleurosaurs, *Palaeopleurosaurus* and *Pleurosaurus*, are recovered as sister taxa but resolution between the remaining core taxa is otherwise poor.

Derived characters that support the inclusion of the Vellberg jaws within Lepidosauria include (character number and coding according to matrix, see Additional file 1): anterior marginal teeth located against a prominent labial wall (pleurodonty): 40(2); the presence of obvious dental regionalisation into anterior and posterior sections: 85(1); posterior marginal teeth with no obvious boundary between tooth and bone: 87(2); anterior marginal teeth with slow tooth replacement (spaces and tooth replacement pits rare, tips may be worn): 88(1); posterior marginal teeth with no evidence of tooth replacement (no spaces, teeth often clearly worn): 89(2); posterior marginal teeth located on the crest of the jaw bone (acrodonty): 90(3); lingual subdental shelf present anteriorly: 91(1); coronoid process of the dentary with some expansion: 93(1). Four of these characters; 87(2), 88(1), 89(2), and 90(3), also secure the jaws within Rhynchocephalia.

### Lepidosauria – phylogenetic topology

**Figure 4:**
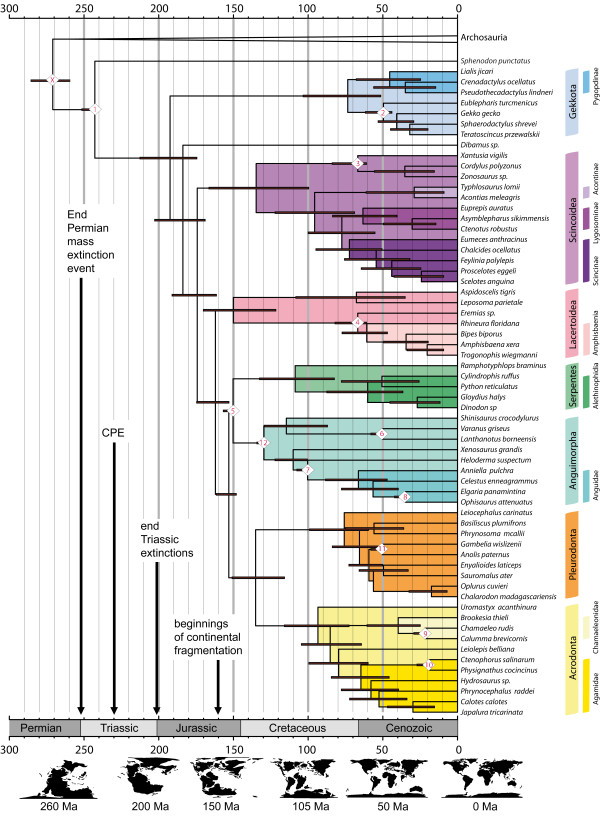
**Maximum clade credibility tree (BEAST) with constrained nodes labelled according to Table**2**.** Tectonic maps were redrawn from Blakey [58]. CPE indicates the Carnian Pluvial Event [61]. Calibrated nodes are numbered X and 1–12 as in Table 1 but Y, crown Archosauria, is not shown. For results from the MrBayes analysis, including posterior probabilities of separate nodes, see Additional file 5.

The topology obtained from our molecular divergence dating in the BEAST analysis (Figure 4: maximum clade credibility tree) is generally the same as that found by previous studies based on molecular data (e.g. [41,51,145]): Lepidosauria is monophyletic, Gekkota is the sister taxon to all other Squamata, amphisbaenians are nested within Lacertoidea, and Iguania is sister group to Serpentes + Anguimorpha. However, there are there are areas of disagreement some with two recent major studies: Townsend et al. [146] and Pyron et al. [2].

Townsend et al. [146], focus on phylogenetic relationships within iguanians using a greater number of taxa (47 vs 20) and additional genetic data (29 gene regions vs 1). In contrast to our analysis, this study recovers chamaeoleons as the sister taxon to all remaining acrodontans (including *Uromastyx*). Also, apart from a *Chalarodon* + *Oplurus* clade there are notable differences in the arrangement of the pleurodont iguanians. However, the interrelationships of the pleurodont taxa used here (and the clades they represent) remain problematic even in this larger analysis.

Pyron et al. [2] employ a “supermatrix approach” to include 4161 squamatan taxa with data from 12 genes. The supermatrix has unfortunately a very large amount of missing data, 81%. The study confirms most of the previous topologies, but also finds some new arrangements. The main difference between its results and those of the present study is they recover Serpentes as sister to a clade comprising Iguania + Anguimorpha. However, the support for this grouping is moderate (79% bootstrap support).

### Lepidosauria – molecular divergence dating

For the divergence time between Lepidosauromorpha (Lepidosauria plus stem group) and Archosauromorpha (Archosauria plus their stem group), our analysis provides a median date of 271 Mya (259–285), which is close to the boundary between the Early (Cisurian) and Middle (Guadalupian) Permian. For crown-group Lepidosauria we recover a date of 242 Mya (238–249.5) and for crown-group Squamata a date of 193 Mya (176–213.2). Dates of origin for all major squamate clades (Gekkota, Scincoidea, Lacertoidea, Serpentes, Anguimorpha, Pleurodonta, and Acrodonta) lie within the Mesozoic (Tables 2 and 3, Figure 4). Only Gekkota and Pleurodonta possess credibility intervals that extend into the Cenozoic. Of these major clades, most have a median estimated date within the Cretaceous except for Lacertoidea which is in the Late Jurassic. Within Iguania, the most recent common ancestor of Acrodonta and Pleurodonta is estimated to have existed in the Early Cretaceous (135 Mya) whereas the clade of *Oplurus cuvieri* + *Chalarodon madagascariensis* is estimated to have appeared no more than 33 Mya. The origin of Alethinophidia and Amphisbaenia both lie close to the K-Pg boundary (66 Mya).

**Table 3:** Dates for the most recent common ancestor of major nodes in the lepidosaur phylogenetic tree

**Group**	**Median**	**95% HPD lower**	**95% HPD upper**
Crown Lepidosauria (lizard-tuatara)	242.0	238.0	249.5
Crown Squamata	193.0	176.0	213.2
Crown Gekkota	76.2	52.4	101.0
Crown Scincoidea	137.6	107.3	168.7
Crown Lacertoidea	150.0	116.4	190.7
Crown Serpentes	109.6	81.1	137.0
Crown Anguimorpha	129.5	128.1	134.2
Crown Iguania	135.8	116.7	152.0
Crown Pleurodonta	75.8	59.6	97.8
Crown Acrodonta	96.0	73.9	121.9

These divergence estimates were calculated using the uncorrelated lognormal relaxed clock model in BEAST.

## Discussion

### Local palaeoecological implications

In addition to a recently discovered procolophonid jaw [84], the Vellberg rhynchocephalian material represents the first small vertebrate remains from the source locality. It is generally agreed that *Diphydontosaurus*-like rhynchocephalians fed on small invertebrates [15,91,138,147-149]. This is supported by their general body size, tooth shape and build of the lower jaw. The teeth are the same shape as tools that can puncture soft materials with relative ease, but are vulnerable to extreme torsion and bending [138,147]. The slender jaws provide long out-levers for rapid closure and capture of small active prey but are not suited to withstanding substantial loading forces [149,150]. The morphology of the Vellberg rhynchocephalian is consistent with these attributes, which is noteworthy as a predator of small invertebrates has not previously been described from this locality. By contrast, all of the taxa currently known from Vellberg (at least as adults) are suited to feeding on small vertebrates or fish. In turn, the Vellberg rhynchocephalian would itself have been prey for other animals in the community, such as immature individuals of *Batrachotomus* and other archosauromorphs. Like small vertebrates in modern communities (e.g. [151]), the lepidosaurs were probably an important link in the food chain between primary and tertiary consumers.

### Global importance of the locality

The Middle Triassic record of small gracile vertebrates is poor. There are several rock units from around world that preserve terrestrial vertebrate remains: the Manda beds of Tanzania, Africa (e.g. [152-154]); the oldest part of the Santa Maria Formation (Fm) of Brazil (e.g. [155,156]); the Chañares Fm of Argentina (e.g. [157]); the Moenkopi Fm of North America [158,159]; the Yerrapalli Beds of India (e.g. [160,161]); the upper part of the Beaufort Group of the Karoo Basin (e.g. [162,163]); the Kelamayi Fm, Ermaying Fm, and Hongyanjing Fm of China (e.g. [164,165]); the Donguz and Bukobay of Russia (e.g. [64,166]); the Omingonde Fm of Namibia [167]; part of the Fremouw Fm of Antarctica [168]; the Zarzaïtine Series of Algeria [169]; the Areniscas y Lutitas del Figaro unit of Spain [170]; and a few units in the United Kingdom such as the Otter Sandstone (e.g. [142,143]). However, fossils from the associated localities typically represent medium or large vertebrates such as trematosaurids, rhynchosaurs, cynodonts, and early archosaurs (e.g. [154,160,165]). Animals of small size (skull length <30 mm long) such as procolophonid reptiles are occasionally recovered but these are typically robust remains (e.g. [142,155]). Therefore, as a new microvertebrate locality, Vellberg is expected to provide a more balanced picture of the Middle Triassic fauna and palaeoecological communities.

Vellberg may also shed light on the early fossil record of important tetrapod groups such as frogs, salamanders, caecilians, albanerpetontids, and choristoderes. All of these groups should have representatives in the Middle Triassic but currently none are known (e.g. [171-178]). Whether this absence of data is related to a failure to sample appropriate facies or a tendency for these animals to be small and gracile, or both, remains unclear [171]. Nevertheless, the material described here demonstrates that Vellberg has the potential to yield remains of other small tetrapods and to provide important information on a poorly known period of significant change in global ecosystems.

### Divergence estimates and congruence with the fossil record

As an independent test of the internal ages of Squamata, we compared them to eight well described and dated fossils that could have been used as additional age constraints. All of them support our dating (Additional file 6), being as old or older than the mean of the estimate. However in three cases they would have truncated the younger bound of the credibility intervals by about 10 Mya.

Beside the manual control of eight alternative calibrations, we also ran the fossil-based cross validation analysis implemented in the penalized likelihood (PL) method of Near and Sanderson [131] on the 14 fossils used (Additional file 6). Simplified, the cross validation procedure sequentially removes one fossil at a time and estimates the node it constrains, to test whether a fossil causes a significant shift towards an older age of the node. Although this is not necessarily a problem with well described fossils, it may indicate a significant rate change close to that node that needs to be calibrated. Cross validation of our data set indicates that the most influential fossil is the calibration of crown-group Diapsida CNY (increased by 52 Mya, fraction score 0.24). This result is not unexpected as it is the node that constrains the root of the phylogeny (Additional file 6). The only other fossil that increases the age estimate significantly in the PL analysis is the fossil constraining the crown-group Anguimorpha CN12 (Figure 4), with an increase of ~8.6 Mya and a fraction score of 0.07.

### Prior distribution of fossil-constrained ages

The setting of prior distributions for constrained ages is a non-trivial task. For the final dating analysis we used an exponential prior calibration density on divergence times. This approach means that the likelihood for the age of a node is highest at the age of the fossil, whilst the older possible ages have lower likelihood. Statistically the first ancestor of a lineage is not the oldest fossil known or recognisable clade member based on clear autapomorphies [179]. Therefore the use of the exponential prior is suboptimal, and the inferred ages are likely to be more prone to underestimation compared to other alternative priors. The widely used lognormal prior (see e.g. [39,43]) implies that it is more likely for the real age to be older than the fossil. It can be argued that this prior would better represent the ghost lineage that must exist. However, in most cases there is no objective way of choosing the shape of the prior distribution, especially not in the case of organisms that are rarely preserved as fossils, and the analysis would potentially be highly biased toward the authors’ opinion on the fossil ghost range. Even if the lognormal prior could potentially approximate the true ages better, we chose the exponential prior because the minimum age of the fossil constraint is the only known date, the prior will be less biased toward the opinions of the researchers, and it represents a sound method from a philosophical viewpoint; our hypothesis is easily falsified if older fossils are found.

For comparison we also ran the analysis with less informative priors, such as a uniform prior with minimum age from the fossil and maximum age 50 million years older, which is probably too old for at least most of the constrained nodes. This approach tended to push all nodes towards older ages, resulting in median ages close to the given maximum age. It seems unlikely that all fossil records have a “ghost range” of almost 50 million years, so we do not recommend this prior distribution unless there is no other way of constraining the node.

The mean of the exponential prior was set consistently to 4.0 (in absolute ages a possible interval of 15–20 Mya with a small probability of estimated dates being older) to provide a plausible and hopefully useful dating of the lepidosaur and squamate divergence times. As a comparison we ran analyses with mean 5.0 and 6.0 (intervals of about 20 and 25 Mya, respectively). In general the resulting ages were within the range of ±5 My compared to the analysis with mean 4, but in some cases with larger credibility intervals (data not shown).

To check the influence of the highly informative priors on ages and monophyly, we also ran the same analysis but with sampling from priors only (by creating an empty alignment). The topology obtained was different, despite monophyly enforced on 16 nodes (beside the nodes constrained by fossil ages, mammals were set as sister to the reptiles (including Aves), and Squamata was set as monophyletic). The posterior density of ages on constrained nodes was similar for some of the nodes, whilst others had a very different distribution. This indicates that the results were not determined by the prior assumptions alone, but that the sequence data also influenced the results for all nodes.

### Previous age estimates of lepidosaurs and squamates

**Figure 5:**
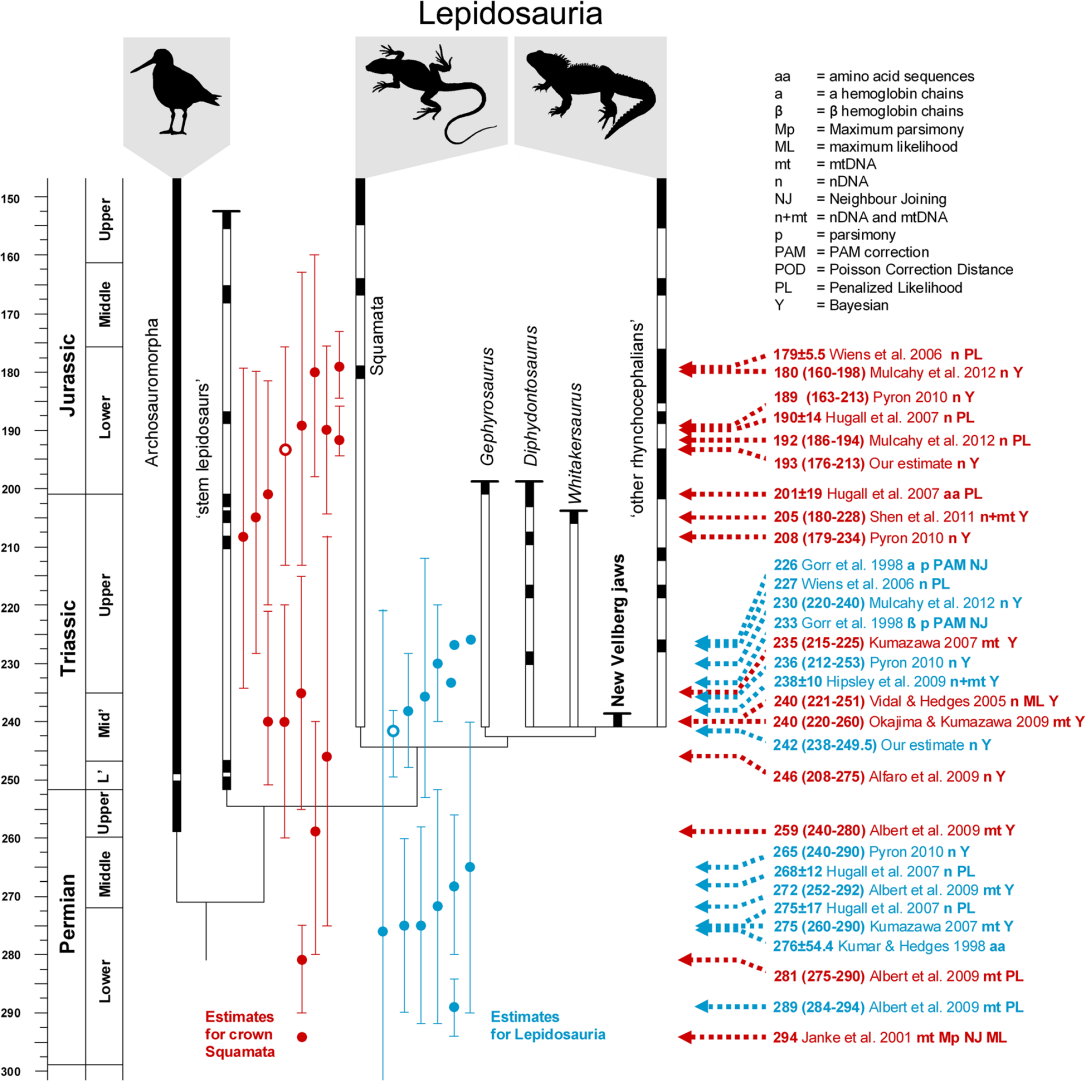
**The phylogenetic relationships and fossil record of early lepidosaurs compared to molecular divergence estimates.** Estimates for the origin of Lepidosauria based on previous molecular studies are listed on the right in blue with short arrows. Estimates for the origin of crown group Squamata are listed on the right in red with long arrows. Timescale based on Gradstein et al. [47]. Fossil records include those described, or referred to, in Butler et al. [105], Carroll [27], Clark and Hernandez [31], Colbert [30], Evans [8,9,26,33,91], Evans and Białynicka [34], Evans and Jones [5], Evans et al., [18], Fraser [22,23,136], Fraser and Benton [11], Heckert et al. [24], Nesbitt [180], Renesto [137], Reynoso [19,150], Robinson [29], Sues and Hopson [13], Sues and Olsen [12], Whiteside [15], and others listed in Evans et al. [181] and Jones et al. [10].

Previous studies attempting to estimate the timing of the origin of Lepidosauria and Squamata have suggested a range of different ages (Table 1, Figure 5). The “outliers”, unusually recent or old dates, may be due to the method used, the number and selection of taxa, choice of molecular marker and of course the choice and number of fossil calibrations.

Gorr et al. [40] used a global clock approach to estimate divergence times within reptiles (including Aves). They concluded that there were large differences in evolutionary rates of reptilian hemoglobins between larger groups, causing an erroneous topology, so their age estimates should be viewed with caution. In a study on vertebrates, Kumar and Hedges [35] estimated gene-specific substitution rates, dated the separate gene trees, and then averaged over the trees to get one dated tree. As rates vary among lineages and therefore do not obey a global clock, they first excluded genes with extensive heterogeneity, and excluded the outliers before averaging over gene trees. Despite this, all nodes closer to the root showed large ghost ranges between estimates and first fossil record (e.g. Agnatha originating in the Precambrian), which is likely due to the method being unable to distinguish between extensive time or fast substitution rates. They conclude that the molecular ages are not overestimating the divergences, and that there are substantial gaps in the fossil record. Their estimate for Lepidosauria was 276±54.4 Mya.

Janke et al. [48] used mitochondrial genomes of a total 35 species to obtain rough estimates of divergence times for Squamata and turtles, assuming a constant evolutionary rate between 2 reference points: the Synapsida/Diapsida split (310 Mya) and the Crocodylidae/Aves split (254 Mya). Previously published genomes of a snake and side-necked turtle were excluded on the basis that their fast evolutionary rates complicate the phylogenetic analysis. The phylogeny and dating only contained two squamates (*Iguana* and the mole skink *Eumeces*). Neverthless, the origin of “Squamata” is stated to be 294 Ma and this date is argued to be consistent with the fossil record. However, this value actually represents the estimated divergence time between the lineage that includes the two squamates from one that includes turtles, crocodiles, and birds (*Sphenodon* was not used). Therefore this estimate more correctly represents the divergence time of Lepidosauromorpha rather than Squamata.

In general, studies using the Bayesian “multidivtime” method [182] give older age estimates than our study [37,38,49-51]. This is most likely an artifact of the method, which due to a strong autocorrelation assumption (the “minab” prior) tends to smooth ages towards the root of the tree to be consistent with the greatest tree depth (e.g. [36,183]). This bias is also more prominent in studies with few taxa, and in the studies listed above the number of squamates range between 19 and 38. In practical terms this means that most of the employed minimum age constraints towards the leaves are uninformative in these “multidivtime” analyses. For the age of Lepidosauria we find no overlap between our deepest credibility interval (251.4 Mya) with the shallowest confidence intervals of Kumazawa [37] or Albert et al. [38], and in all the above mentioned studies using multidivtime crown-group Squamata is estimated to be Triassic or older.

Wiens et al. [41] used the semi-parametric penalized likelihood (PL) method of Sanderson [102] and 11 fossil constraints. For the PL analysis it is necessary to set a fixed age close to the root. The focus of the study was the internal divergences in Squamata, and they therefore chose to use the oldest known rhynchocephalian fossil to fix the most recent common ancestor of Squamata and Rhynchocephalia to 227 Mya. This approach may have caused an underestimation of the age of crown-group Squamata (178.7 Mya compared to 193.1 in this study). Hugall et al. [36] used RAG1 sequence and the PL method to study tetrapod diversification, including a total of 35 squamates. They used a maximum age of 450 Mya for the lungfish-tetrapod root and tested different sets of calibrations for internal nodes. All employed constraints were fixed, to avoid the method artefact of “model overfitting”, meaning that constraints closer to the leaves can lead to overestimation of deeper nodes. They estimated the split median ages between *Sphenodon* and Squamata to be 250–275 Mya, and that of crown-group Squamata to be 171–201 Mya, depending on the calibration scheme employed.

Hipsley et al. [42] used the same constraint for the lizard-tuatara split as Wiens et al. [41] but the former used a Bayesian probabilistic method as implemented in the TreeTime software [184]. To account for the uncertainty in fossil calibrations and the likelihood of the true age of a node being older than the first fossil record, the age constraint was set with a hard upper bound of 228 Mya and soft lower bound of 239.4 Mya. Their estimate for the *Sphenodon*-Squamata split was 238±10 Mya.

Pyron [39] proposed a method that can objectively test fossil placement and the likelihood of age estimates by comparisons between datasets of different studies. The empirical example is divergence analyses on RAG-1 DNA from 129 gnathostome taxa to compare the affect of two different sets of fossil calibrations. The sample included *Sphenodon* and 44 squamates. The uncorrelated lognormal method in BEAST was used, and a lognormal distribution was chosen for the prior distribution of ages from the fossil calibrations. Four fossil calibrations from Müller and Reisz [52] provided a mean estimate of 236 Mya (credibility interval 212–253) for Lepidosauria and 189 Mya (163–213) for Squamata whereas five fossil calibrations from Hugall et al. [36] provided a mean estimate of 265 Mya (240–290) for Lepidosauria and 208 Mya (179–234) for Squamata. The shallower estimates were preferred based on a comparison to the wider fossil record. These dates are similar to our own but have greater confidence intervals.

Mulcahy et al. [43] estimated divergence dates for squamates using 64 ingroup species and 25 nuclear loci (19,020 base pairs in total), comparing the results obtained from Penalized Likelihood (r8s) and the uncorrelated lognormal method in BEAST. The overlap between their study and the present one is substantial for terminal taxa. There are however some important differences in the fossil constraints such as the use of a younger rhynchocephalian fossil here (for a detailed comparison see Additional file 1). Mulcahy et al. [43] fixed the topology to the same maximum likelihood tree they used as input in the r8s analysis, to facilitate direct comparisons of ages between PL and BEAST, whilst we only constrained the calibrated nodes to be monophyletic. As opposed to our approach of using exponential age priors, Mulcahy et al. [43] applied lognormal distribution of ages for the 11 fossil constrained internal (Lepidosauromorpha) nodes. The oldest rhynchocephalian was set to 222.8 Mya, based on the Vinita specimen from the Ladinian–Carnian boundary. Note that this age was chosen using the timescale of Gradstein et al. [46] rather than the more recent Gradstein et al. [47]. The lognormal priors were set to have a mean and standard deviation of 1.0 – meaning a very narrow interval (about 3 Mya) with an arbitrary mean close to the minimum age of the fossil (e.g. for Lepidosauria 223.4 Mya, 222.9-225.9). Mulcahy et al. [43] conclude that the BEAST/lognormal clock analysis gives younger ages than the r8s/PL analysis. This is not surprising, considering that the internal priors have soft lower bounds but are strong enough to behave as if they have a hard bound, thereby constraining other internal nodes more than the minimum age constraints in the PL analysis, where the only lower bound is the fixed root. This is also likely to be the reason why the BEAST estimates seem more stable with narrower credibility intervals.

### Origin time of Lepidosauria, crown-Squamata, and other major clades

The Vellberg jaw helps to bridge an important gap in the fossil record and establish that Lepidosauria (stem group Rhynchocephalia, and stem group Squamata) diverged at least 240 Mya (Figure 4). Discovery of lepidosaur remains in the Middle Triassic is consistent with previous predictions made by palaeontologists (e.g. [9,26,34]). It is also consistent with the Late Triassic rhynchocephalian fossil diversity [9,23-25]. This new record from Vellberg supersedes previously used molecular dating calibration points of 223, 227, or 228 Mya for the lizard-tuatara split [37-43,45].

For the divergence between Lepidosauromorpha and Archosauromorpha (bird-lizard split) the median of our estimate, 271 Mya (259–285), lies close to the boundary between the Lower and Middle Permian. This date is deeper than the oldest known fossils of either group (Figure 5): the earliest known archosauromorph is *Protorosaurus* from the Upper Permian (Wuchiapingian) of northeast England (UK) and the Kupferschiefer of Germany [104] and the earliest certain lepidosauromorph is *Sophineta* from the Lower Triassic (Olenekian) of Poland [5,34]. However, the possibility that large gaps in the fossil record remain, particularly so for stem-lepidosaurs (>20 Mya), highlights the need to survey further fossil localities in the Middle and Late Permian for small vertebrates.

Importantly, our estimate strongly suggests that the origin of Lepidosauria postdates the Permian mass extinction event (252 Mya), which represents a significant period of environmental upheaval possibly linked to a runaway green house environment [65,67,68]. An Early-Middle Triassic origin and radiation of Lepidosauria would be associated with general changes from fairly uniform warm-arid environments towards ones experiencing humid-arid fluctuations and monsoon systems [59,61,62,90]. Complex biodiversity was still in the process of reestablishment after the Permian end mass extinction event [65,67,68]. Vegetation in the Middle Triassic was dominated by gymnosperms such as cycads, ginkos and conifers [59,62]. Coeveal macrofaunal changes include the diversification of early archosaurs such as the sail-backed poposaurs and appearance of the first dinosauriformes (e.g. [105,152,180,185]). The subsequent “Carnian Pluvial Event” (CPE) of the Late Triassic [61] is thought to represent a global increase in rainfall and further shifts towards more humid climates (Figure 4).

Our results suggests that the origin of crown-group Squamata lies in the Early Jurassic,190 Mya (175–212). We cannot exclude the possibility that crown-squamates appeared before the late Triassic extinctions but our median estimate post-dates them. Our estimate lies soon after changes in general vegetation that indicate changes towards warmer climates and greater continental aridity [54,62]. This may be part of a general shift towards more regionalised climates and environments, at least in the northern hemisphere. The earliest secure lizard fossils currently referred to crown-Squamata are Middle Jurassic in age and therefore do not conflict with our estimate [8,17,18].

The Cretaceous origin of most major crown-groups suggests the radiation of Squamata occurred after and alongside continental fragmentation (Figure 4, Table 3, [58,186]. Therefore the widespread distribution of many modern lizard groups today (e.g. [1]) probably requires a number of post Jurassic dispersal events to have occurred. Evidence that transoceanic dispersal of squamates is possible does certainly exist (e.g. [187-190]) and the distances between continental fragments in the second half of the Mesozoic were much less than they are today [191].

Our estimates for the origin of most modern groups coincide with a general improvement of the squamate fossil record [5-8] and contraction of rhynchocephalian distributions to southern continents [10,20,181,192]. This shift in lepidosaur communities may be related to expansion/contraction of preferred environments [6,7,21] or displacement by active competition [20,192,193], but distinguishing between the two hypotheses remains problematic [5,25]. The Early Jurassic to Early Cretaceous diversification of crown-group squamates is concurrent with that of several modern lineages of beetles [54,56]. However, rather than reflecting a predator–prey relationship it may be symptomatic of the general development of more modern ground cover and microhabitats.

The divergence estimates for both crown-group Iguania, 136 Mya (117–152), and total group Iguania 153 Mya (148–161) post date the fossil taxon *Bharatagama* from India originally referred to Iguania [18]. It is possible that *Bharatagama* represents an early stem crown-group squamate with a jaw morphology convergent with modern acrodont iguanians, or that it belongs to another clade.

Our estimated origin time for Gekkota, 76 Mya (52–101), is younger than that of some previous studies but there is some overlap between credibility intervals (e.g. [36,43,194]). There are also two early-mid Cretaceous fossils that could potentially challenge our crown-group age of Gekkota: *Cretaceogekko burmae* preserved in amber from Myanmar (>97.5 Mya) [195] and *Hoburogekko suchanovi* from Mongolia (125–99.6 Mya) [196,197]. Both fossils likely belong to the gekkotan lineage but their precise relationship with extant geckos is unclear [197]. *Cretaceogekko* was described as crown-group gekkotan based on it’s advanced adhesive toe pads, but it has recently been inferred that specialized toe pad morphology has evolved (and been lost) several times across the gekkotan phylogeny [198]. Hence it is not possible from morphological characters alone to determine crown-group affinity. The redescription by Daza et al. [197] of *Hoburogekko* concludes that the combination of jaw and skull characters is likely to belong to a stem-group gekkotan, and that a phylogenetically conservative placement of these Cretaceous fossils is recommended.

## Conclusions

Using the age of a new lepidosaur fossil from the Middle Triassic of Germany and 13 other fossil constraints, we estimate that Lepidosauria originated between 238 and 249.5 Mya (median age 242) in the Early-Middle Triassic, and importantly that their origin and diversification occurred after the end-Permian mass extinction rather than before it. This date is consistent with previous estimates inferred using fossil data such as that made by SE Evans ([26]: page 407). We also estimate crown-group Squamata originated between 175 and 212 Mya (median age 193) in the Late Triassic-Early Jurassic concurrently with notable shifts in vegetation, fauna, and climate. However, the precise relationship between the appearance of crown-group squamates and the end Triassic mass extinction remains uncertain. The origins of most major squamate clades such as Anguimorpha and Acrodonta occurred in the Late Jurassic and Cretaceous, taking place during and after continental fragmentation. Therefore, oceanic dispersal is likely to have been an important factor in the global radiation and evolution of squamates.

Molecular datings are an important part of evolutionary biology, and thousands of studies including dated phylogenies have been published in the last few decades. Several studies have shown that an increased number of taxa and, more importantly, correctly assigned fossil constraints improve datings. There is however no consensus about which methods provide the most reliable results, and for the Bayesian methods the priors on node ages (as well as priors affecting e.g. topology) are highly debated. All molecular datings are open for refinement, and the estimates given here for the origin of Lepidosauria and Squamata will probably be superseded. As the only extant rhynchocephalian, *Sphenodon* represents the best available sister taxon for molecular analysis. Nevertheless, it is taxonomically isolated: the end member of a very long branch. Large credibility intervals will persist around the divergence date of crown-group Squamata in the absence of fossils that can be reliably placed around this node. Until then, the new lepidosaur fossil described in this study will play an important part in future divergence estimate analyses in early lepidosaur history.

### Note added post-acceptance

Renesto & Bernardi [199] recently re-attributed *Megachirella* to Lepidosauromorpha on the basis of a new phylogenetic analysis.

## Availability of supporting data

The matrix is also deposited in the Dryad data repository (http://datadryad.org/), with the Digital Object Identifier (DOI) of http://dx.doi.org/10.5061/dryad.gr573.

## Competing interests

The authors declare we have no competing interests.

## Authors’ contributions

MEHJ carried out the morphological comparisons, CT data processing, Scanning Electron Microscopy, camera lucida drawings, initial calibration point evaluation, drafted the manuscript, and contributed to the morphological phylogenetic analysis. CAH collected the genetic sequence data and performed the sequence alignment. CLA carried out the molecular dating analyses and final morphological phylogenetic analysis. SEE contributed to morphological comparisons, initial identification of calibration points, and the morphological phylogenetic analysis. JM evaluated calibration points. RRS carried out the field work and prepared the specimens. MEHJ and CLA wrote the paper. All authors reviewed, edited, and approved the final manuscript.

## Supplementary Material

Additional file 1: Summary of previous molecular divergence estimates, further details of the morphological phylogenetic analysis, and calibration points employed. Click here for file

Additional file 2: A movie of a surface model of SMNS 91060 based on CT data. Click here for file

Additional file 3: A movie of a surface model of SMNS 91061 based on CT data. Click here for file

Additional file 4: List of sequences used from GenBank for 77 extant amniote taxa. Click here for file

Additional file 5: Majority rule consensus tree from MrBayes, phylogram with posterior probabilities shown. Click here for file

Additional file 6: **Results of the fossil cross-validation using r8s (see [**[131]**]).** Click here for file
