# Supplementary material for: Integration of molecules and new fossils supports a Triassic origin for Lepidosauria (lizards, snakes, and tuatara)
Source: BMC Evol Biol. 2013 Sep 25;13:208. doi: 10.1186/1471-2148-13-208 (PMC4016551; doi:10.1186/1471-2148-13-208)
Supplement: Additional file 1: — Summary of previous molecular divergence estimates, further details of the morphological phylogenetic analysis, and calibration points employed. [file 1471-2148-13-208-S1.docx]

# SUPPLEMENTARY MATERIAL

**Previous estimates**

Table S1.1 Previously published dates for the origin of Lepidosauria (tuatara-lizard divergence).

| Reference | Date (Mya) | Context |
| --- | --- | --- |
| Evans 1991 | “By the end of the early Triassic” [247] | Inferred from the fossil record. |
| Gorr et al. 1998 | 230 | Inferred from the fossil record as discussed in Benton 1985, 1990 and Evans 1988. Used as a calibration point. |
| Gorr et al. 1998 | 233 | Date calculated from rate changes in α hemoglobin chains. Rate calibrated using tuatara-lizard divergence (230 Mya) and three divergence dates of clades within snakes. |
| Gorr et al. 1998 | 226 | Date calculated from rate changes in β hemoglobin chains. Rate calibrated using tuatara-lizard divergence (230 Mya) and three divergence dates of clades within snakes. |
| Kumar and Hedges 1998 | 276±54.4 | Estimated from gene-specific substitution rates in separate gene trees averaged over the trees (after exclusion of outliers and genes with extensive heterogeneity) |
| Evans 2003 | “Early-Middle Triassic”  [= 235-252] | Inferred from the fossil record. |
| Rest et al. 2003 | 230 | Inferred from the fossil record based on Benton (1993) and Carroll (1988). Used as a calibration point. |
| Wiens et al. 2006 | 227 | Inferred from the fossil record based on Sues and Olsen 1990. Used as a calibration point. |
| Kumazawa, 2007 | 260 – 290 | Molecular divergence date obtained from a Bayesian autocorrelated clock “multidivtime”, using mitochondrial DNA and four fossil constraints including a lizard-bird divergence date of 255-305. |
| Hugall et al. 2007 | 268 – 275 | Molecular divergence date obtained using a RAG-1 phylogeny obtained by MrBayes and five fossil calibration dates spread around the tetrapod tree including 99 Mya for the *Heloderma-Elgaria* split, 245 Mya for the bird-crocodile divergence date and 315 Mya for the origin of Amniota. Results obtained from penalized likelihood (r8s) were 268 ± 12 based on nucleotides and 275 ± 17 based on amino acids. |
| Hipsley et al. 2009 | 238 ± 10 | Several clock models were compared using the Bayesian TreeTime software and the uncorrelated lognormal model was found to best fit the  data. Median date obtained using a mixture of mitochondrial and nuclear genes and a minimum constraint of 228 Mya (inferred from the fossil record of Sues and Olsen 1990). |
| Jones et al. 2009 | 240 – 250 | Inferred from the fossil record. |
| Albert et al. 2009 | 272 (255-290) | Molecular divergence estimates based on complete mitochondrial genomes and penalized likelihood. A date of 227 Mya used as a minimum age for the origin of Lepidosauria based on Sues and Olsen (1990). |
| Albert et al. 2009 | 289 (282-293) | Molecular divergence estimates based on complete mitochondrial genomes and Bayesian autocorrelated clock “multidivtime”. A date of 227 Mya used as a minimum age for the origin of Lepidosauria based on Sues and Olsen (1990). |
| Pyron 2010 | 236 (212-253) | BEAST, Bayesian uncorrelated lognormal clock using 4 fossil constraints from Müller and Reisz (2005). |
| Pyron 2010 | 265 (240-290) | BEAST, Bayesian uncorrelated lognormal clock using 5 fossil constraints from Hugall et al. (2007). |
| Mulcahy et al. 2012 | ~233 (223-243) | BEAST, Bayesian uncorrelated lognormal clock (discussed further below) |
| Mulcahy et al. 2012 | ~275 (na) | r8s, Penalized Likelihood (discussed further below) |

Table S1.2 Previously published dates for the origin of Squamata (shared common ancestor of all snakes and lizards).

| Reference | Date (Mya) | Context |
| --- | --- | --- |
| Janke *et al*. 2001 | 294 | Rate changes in mitochondrial DNA |
| Vidal & Hedges 2005 | 240  (221–251) | Inferred from the fossil record. Molecular clock date obtained from Bayesian autocorrelated clock “multidivtime”, nuclear gene data and a constraint bracket of 200 - 251 Mya based on the fossil record. |
| Wiens *et al*. 2006 | 178.7 ± 5.5 | Molecular clock date obtained using the RAG-1 nuclear gene and 11 calibration dates including 228 Mya for the tuatara-lizard split (inferred from the fossil record based on Sues and Olsen 1990). |
| Hugall et al. 2007 | 190 - 201 | Molecular clock date obtained using a RAG-1 phylogeny obtained by MrBayes and five fossil calibration dates spread around the tetrapod tree including 99 Mya for the *Heloderma-Elgaria* split, 245 Mya for the bird-crocodile divergence date and 315 Mya for the origin of Amniota. Results obtained from penalized likelihood (r8s) were 190 ± 14 based on nucleotides and 201 ± 19 based on amino acids. |
| Alfaro et al. 2009 | 246 (208 -275) | BEAST, Bayesian uncorrelated lognormal clock, multiple calibration points with lognormal distribution and hard younger bounds. |
| Albert et al., 2009 | 259 (240–278) | Molecular clock estimates based on complete mitochondrial genomes and penalized likelihood. A date of 227 Mya used as a minimum age for the origin of Lepidosauria based on Sues and Olsen (1990). |
| Albert et al., 2009 | 281 (289–299) | Molecular clock estimates based on complete mitochondrial genomes and Bayesian autocorrelated clock “multidivtime”. A date of 227 Mya used as a minimum age for the origin of Lepidosauria based on Sues and Olsen (1990). |
| Okajima & Kumazawa 2009 | 240 (220-260) | “multidivtime”, Bayesian autocorrelated clock |
| Pyron 2010 | 189 (163-213) | BEAST, Bayesian uncorrelated lognormal clock using 4 fossil constraints from Müller and Reisz (2005). |
| Pyron 2010 | 208 (179-234) | BEAST, Bayesian uncorrelated lognormal clock using 5 fossil constraints from Hugall et al. (2007). |
| Shen et al. 2011 | 205 (180-228) | “multidivtime”, Bayesian autocorrelated clock. Three nodes constrained using dates from Müller and Reisz (2005) and Benton and Donoghue (2007). |
| Mulcahy et al. 2012 | 180 (160-198) | BEAST, Bayesian uncorrelated lognormal clock (discussed further below) |
| Mulcahy et al. 2012 | 191.8 (186-194) | r8s, Penalized Likelihood (discussed further below) |
|  |  |  |

Table S1.3 Previously published dates for the divergence of Lepidosauromorpha and Archosauromorpha (bird-lizard split).

| Reference | Date (Mya) | Context |
| --- | --- | --- |
| Reisz and Müller 2004 | 252–257 | Proposed calibration date inferred from the fossil record. |
| Benton & Donoghue 2007 | 259.7–299.8 | Proposed calibration date inferred from the fossil record. |
| Hugall et al. 2007 | 285–289 | Molecular clock date obtained using the RAG-1 nuclear gene and five fossil calibration dates spread around the tetrapod tree including 99 Ma for the *Heloderma*-*Elgaria* split, 245 Mya for the bird-crocodile divergence date and 315 Mya for the origin of Amniota. 285 ± 11 Mya obtain with nucleotide Bayesian analysis and 289 ± 13 Mya obtain with an amino acid Bayesian analysis. |
| Sanders and Lee 2007 | 282.8 (263–303.9) | Estimate using a relaxed clock analysis with lognormal calibration points. |
| Sanders and Lee 2007 | 261.1 (249.5–269.1) | Estimate using a global clock analysis. |
| Alfaro et al. 2009 | 270 (257–292) | Node used as a calibration point with reference to Müller and Reisz (2005) and Reisz et al. (2007).: 255 Mya with a soft upper bound set to 282 Mya. |
| J Müller in San Mauro 2010: 556 | 271–259.7 | Proposed calibration date inferred from the fossil record. |

**Morphological cladistic analysis**

**A data matrix of** 22 taxa and 100 characters was compiled.

Taxon sample

Of the 22 taxa used, 7 represent outgroup taxa and 15 represent ingroup taxa. Although *Paliguana* and *Saurosternon* were once considered to represent lepidosauromorphs or even ‘true lizards’ (within Squamata) (Carroll 1975, 1977) this is not supported by subsequent studies and cladistic analyses (Evans, 2003).

Kuehneosauridae (*Kuehneosaurus*, *Kuehneosuchus*, *Icarosaurus*) are generally regarded to be lepidosauromorphs (e.g. Rieppel 1994; Evans 2003; Jones 2008). This is supported by some phylogenetic studies (e.g. Evans 1988, 2009; Gauthier et al. 1988; Lee 2001). However, as Evans (1984) suggested, it is possible they instead represent a clade outside Sauria (Lepidosauromorpha + Archosauromorpha) that exhibits convergence on lepidosaur morphology (Benton 1985; Müller 2003, 2004; Hill, 2005). Whether sauropterygians and turtles are closely related to Lepiodosauromorpha remains uncertain (e.g. Lee 1993; deBraga and Rieppel 1996, 1997; Kumazawa and Nishida 1999; Zardoya and Meyer 1998, 2001; Müller 2003, 2004; Hill, 2005; Werneburg and Sánchez-Villagra 2009; Lyson et al. 2010, 2011; Crawford et al. 2012) but this problem will not be dealt with here.

Squamata was used as a metataxon because the early fossil record of this group remains very poor. Modern examplar taxa were not used to represent Squamata because within this diverse group it is currently uncertain which taxa best represent the plesiomorphic condition.

Outgroup taxa:

*Petrolacosaurus* (Reisz 1977)

*Youngina* (Gow 1975)

*Czatkowiella* (Borsuk-Białynicka & Evans 2009)

*Prolacerta* (e.g., Camp 1945ab Gow 1975; Modesto and Sues 2004)

*Paliguana* (Carroll 1975)

*Saurosternon* (Carroll 1975)

Kuehneosauridae (Robinson 1962, 1967; Colbert 1966; Evans 2009)

Ingroup taxa:

*Marmoretta* (Evans 1991; Waldman and Evans 1994)

*Sophineta* (Evans & Borsuk-Białynicka 2009)

*Squamata* (e.g. Evans 2008)

*Gephyrosaurus* (Evans 1980, 1981ab, 1983; 1985)

*Diphydontosaurus* (Whiteside 1986)

*Vellberg jaw* NEW SPECIMEN

*Whitakersaurus* (Heckert et al. 2008)

*Planocephalosaurus* (Fraser 1982; Fraser and Walkden 1984)

*Rebbanasaurus* (Evans et al. 2001)

*Clevosaurus hudsoni* (Fraser 1988; Jones 2006)

*Palaeopleurosaurus* (Cocude-Michel 1963; Carroll 1985; Carroll and Wild, 1994)

*Pleurosaurus* (Cocude-Michel 1963; Carroll 1985; Carroll and Wild, 1994; Dupret 2004)

*Kallimodon* (Cocude-Michel 1963)

*Priosphenodon* (Apesteguía and Novas 2003)

*Sphenodon* (Günther 1867; Evans 2008; Jones 2008; Jones et al., 2009)

Characters used in the phylogenetic analysis

Many of the characters used have a long history and date back to studies made by Evans (1980, 1988), Whiteside (1986) and Gauthier *et al.* (1988). Others characters include those that were added and modified during a number of subsequent studies (Fraser and Benton 1989; Wu 1994; Sues et al. 1994; Reynoso 1996, 1997, 2000; Reynoso and Clark 1998; Apesteguía and Novas 2003; Dupret 2004). Characters 1 to 77 broadly correspond to those used by Evans (2009) and Evans and Borsuk-Białynicka (2009). These were in error said to be listed in Waldman and Evans (1994) but in actuality refer to part of the analysis that was removed prior to publication.

Despite the number of characters used in previously analyses, this matrix should be treated as new because several characters have been modified in order to accommodate both rhynchocephalians and non-crown lepidosauromorphs.

1: Lacrimal size; 0 = Extensive exposure on the cheek; 1 = Small, confined to orbital rim; 2 = Absent (Evans and Borsuk-Białynicka 2009: ch. 1; Apesteguía and Novas 2003: ch. 9; Reynoso 1996: ch. 6; Wu 1994: ch. 3; Sues et al. 1994: ch. 3).

2: Width of exposed nasal; 0 = Exceeds greatest width of both nares; 1 = Less than the width of both nares (Evans and Borsuk-Białynicka 2009: ch. 2).

3: Frontoparietal external suture seam; 0 = W shaped or crenulated and narrower than nasofrontal suture; 1 = Generally straight and wider than nasofrontal suture (potential for mesokinesis) (Evans and Borsuk-Białynicka 2009: ch. 3).

4: Parietal foramen; 0 = Within parietal; 1 = Frontoparietal border; 2 = Absent (Evans and Borsuk-Białynicka 2009: ch. 4; similar to Apesteguía and Novas 2003: ch. 19 Reynoso 1996: ch. 13; Wu, 1994: ch. 10; Sues et al.: 1994, ch. 10 but anterior edge of the UTF (upper temporal fenestrae) are not used as a landmark for differentiating two states).

5: Postparietals; 0 = Present; 1 = Absent (Evans and Borsuk-Białynicka 2009: ch. 5).

6: Tabulars; 0 = Present; 1 = Absent (Evans and Borsuk-Białynicka 2009: ch. 6).

7: Postorbital-postfrontal relationship; 0 = Postorbital overlies postfrontal; 1 = Postfrontal overlies postorbital (Evans and Borsuk-Białynicka 2009: ch. 7).

8: Squamosal anterior process (in lateral view); 0 = Extends less than halfway above LTF (lower temporal fenestra); 1 = Extends over halfway above LTF and come close to or contact jugal below postorbital (Evans and Borsuk-Białynicka 2009: ch. 8).

9: Quadratojugal anterior process; 0 = Present; 1 = Absent (Evans and Borsuk-Białynicka 2009: ch. 9).

10: Jugal posteroventral process; 0 = Firmly sutured to quadrate or quadratojugal; 1 = Meets quadrate or quadratojugal; 2 = Long process extending at least halfway under the LTF but does not meet quadrate or quadratojugal; 3 = No process or small flange (modified from Evans and Borsuk-Białynicka 2009: ch. 10; Apesteguía and Novas 2003: ch. 12; Reynoso, 1996, ch. 15; Wu, 1994, ch. 18; Sues et al., 1994, ch. 16. Modified from a simple two state presence or absence character to reflect the discussion in Jones 2006b).

11: Quadratojugal; 0 = Always present as separate element at some point; 1 = Never present as separate element (Evans and Borsuk-Białynicka 2009: ch. 11).

12: Supratemporal; 0 = Present; 1 = Absent (Evans and Borsuk-Białynicka 2009: ch. 12; Apesteguía and Novas 2003: ch. 31; Sues et al. 1994: ch. 9; Reynoso 1997: ch. 45).

13: Supratemporal position; 0 = Superficial position; 1 = lies deep in association with ventral face of the postparietal process (Evans and Borsuk-Białynicka 2009: ch. 13).

14: Squamosal ventral process; 0 = Present; 1 = Absent (Evans and Borsuk-Białynicka 2009: ch. 14).

15: Adductor chamber; 0 = Small, quadrate does not extend well below level of occipital condyle; 1 = Large, enlarged adductor fossa and quadrate extends well below adductor fossa (Evans and Borsuk-Białynicka 2009: ch. 15).

16: Nares; 0 = Paired; 1 = Confluent (Evans and Borsuk-Białynicka 2009: ch. 16).

17: Quadrate in posterior view; 0 = Narrow; 1 = Wide and formed into a conch (Evans and Borsuk-Białynicka 2009: ch. 17; similar to Apesteguía and Novas 2003 but with reversed polarity).

18: Quadrate foramen; 0 = Present; 1 = Absent (Evans and Borsuk-Białynicka 2009: ch. 18).

19: Vomerine teeth; 0 = Present and numerous; 1 = Few or absent (Evans and Borsuk-Białynicka 2009: ch. 19).

20: Teeth on transverse flange of pterygoid; 0 = Present; 1 = Absent (Evans and Borsuk-Białynicka 2009: ch. 20).

21: Abducens canal in basisphenoid; 0 = Absent or incomplete; 1 = Present and complete (Evans and Borsuk-Białynicka 2009: ch. 21).

22: Parasphenoid teeth; 0 = Present; 1 = Absent (Evans and Borsuk-Białynicka 2009: ch. 22).

23: Paraoccipital process contact with quadrate; 0 = Present; 1 = Absent (Evans and Borsuk-Białynicka 2009: ch. 23).

24: Paraoccipital process distal ends; 0 = Not expanded; 1 = Expanded (Evans and Borsuk-Białynicka 2009: ch. 24).

25: Stapes; 0 = Thick and perforated for stapedal artery; 1 = Thin and imperforate; 2 = Columelliform usually imperforate in adults (Evans and Borsuk-Białynicka 2009: ch. 25).

26: Quadrate in lateral view; 0 = Straight; 1 = Bowed (Evans and Borsuk-Białynicka 2009: ch. 26).

27: Postorbital-parietal contact; 0 = Present; 1 = Absent (Evans and Borsuk-Białynicka 2009: ch. 27).

28: Postorbital posterior process; 0 = Does not extend beyond posterior margin of the UTF; 1 = Extends beyond posterior margin of the UTF (Evans and Borsuk-Białynicka 2009: ch. 28).

29: Choanal fossa on ventral surface of the palatine; 0 = Absent; 1 = Present (Evans and Borsuk-Białynicka 2009: ch. 29).

30: Snout; 0 = Short and broad; 1 = Long and narrow (Evans and Borsuk-Białynicka 2009: ch. 30).

31: Premaxillae; 0 = Paired in adults; 1 = Fused postnatally (Evans and Borsuk-Białynicka 2009: ch. 31).

32: Parietals; 0 = Paired in adults; 1 = Fused postnatally (Evans and Borsuk-Białynicka 2009: Apesteguía and Novas 2003: ch. 32; Reynoso 1996: ch. 9; Wu 1994: ch. 5; Sues et al. 1994: ch. 6).

33: Metotic fissure; 0 = Exoccipitals sutured to opisthotic above and below metotic fissure; 1 = Fused only above metotic fissure with metotic fissure extending ventrally into basioccipital; 2 = Metotic fissure subdivided to create a dorsal vagus foramen and a ventral opening for the glossopharyngeal nerve and perilymphatic sac (Evans and Borsuk-Białynicka 2009: ch. 33).

34: Frontals; 0 = Paired in adults; 1 = Fused postnatally (Evans and Borsuk-Białynicka 2009: ch. 34; Apesteguía and Novas 2003: ch. 14; Reynoso 1996: ch. 8; Wu 1994: ch. 4; Sues et al. 1994: ch. 5).

35: Splenial; 0 = Absent; 1 = Present (Evans and Borsuk-Białynicka 2009: ch. 35).

36: Angular (posterior extent); 0 = Extends posterior to articular condyle; 1 = Does not extend posterior to articular condyle (Evans and Borsuk-Białynicka 2009: ch. 36).

37: Angular (dorsoventral extent); 0 = Angular extends more than one third up lateral face of lower jaw; 1 = Angular extends less than one third up lateral face of lower jaw (Evans and Borsuk-Białynicka 2009: ch. 37).

38: Retroarticular process; 0 = Short or absent; 1 = Long and slender (Evans and Borsuk-Białynicka 2009: ch. 38; Reynoso 1996: ch. 25; Wu 1994: ch. 19; Sues et al. 1994: ch. 17; lacks third state used in Apesteguía and Novas 2003: ch. 41).

39: Articular condyle; 0 = Outer one third of articular condyle formed by surangular; 1 = Formed entirely by articular, surangular forming outer outer rim (Evans and Borsuk-Białynicka 2009: ch. 39).

40: Tooth location (anterior marginal teeth) (see also character 90); 0 = In shallow sockets or depressions; 1 = Against a low labial wall that is taller than any lingual wall; 2 = Against a prominent labial wall (pleurodonty); 3 = On the crest of the jaw bone (acrodonty) (similar to Evans and Borsuk-Białynicka 2009: ch. 40; Reynoso 1996: ch. 26; Sues et al. 1994, ch19, Apesteguía and Novas 2003: ch. 43; but modified to include only the anterior teeth and four states used rather than two or three to distinguish tooth location. See also character 90).

41: Neural arch fusion; 0 = Occurs post-natally; 1 = Occurs pre-natally (Evans and Borsuk-Białynicka 2009: ch. 41).

42: Zygosphenes and zygantra; 0 = Absent; 1 = Present (Evans and Borsuk-Białynicka 2009: ch. 42).

43: Caudal autotomy; 0 = Absent; 1 = Present (Evans and Borsuk-Białynicka 2009: ch. 43).

44: Trunk vertebrae; 0 = No accessory articulations; 1 = Accessory articulations (Evans and Borsuk-Białynicka 2009: ch. 44).

45: Trunk intercentra; 0 = Present; 1 = Absent (Evans and Borsuk-Białynicka 2009: ch. 45).

46: Vertebrae; 0 = Amphicoelous notochordal; 1 = Amphicoelous non-notochordal; 2 = Procoelous (Evans and Borsuk-Białynicka 2009: ch. 46).

47: Transverse processes on the trunk vertebrae; 0 = Not elongate; 1 = Short; 2 = Long (Evans and Borsuk-Białynicka 2009: ch. 47).

48: Rib heads; 0 = Some cervical ribs with two distinct heads; 1 = All single headed; 2 = One or more rib with three heads (Evans and Borsuk-Białynicka 2009: ch. 48).

49: Trunk vertebrae with free ribs (lumbar region); 0 = Absent; 1 = Present (Evans and Borsuk-Białynicka 2009: ch. 49).

50: Sacral and caudal ribs; 0 = Fuse to vertebra post-natally; 1 = Fuse pre-natally (Evans and Borsuk-Białynicka 2009: ch. 50).

51: Sternum; 0 = A single rod; 1 = Two large plates fuse near adulthood; 2 = Paired but fuse pre-natally (Evans and Borsuk-Białynicka 2009: ch. 51).

52: Anterior margin of scapulocoracoid fenestrated; 0 = Absent; 1 = Present (Evans and Borsuk-Białynicka 2009: ch. 52).

53: Interclavicle; 0 = Robust; 1 = Gracile (Evans and Borsuk-Białynicka 2009: ch. 53).

54: Entepicondylar foramen of humerus; 0 = Present; 1 = Absent (Evans and Borsuk-Białynicka 2009: ch. 54).

55: Prominent posteromedial process of distal epiphysis of radius; 0 = Absent; 1 = Present (Evans and Borsuk-Białynicka 2009: ch. 55).

56: Relative 3rd and 4th metacarpal length; 0 = 4th > 3rd; 1 = 4th about equal to 3rd; 2 = 3rd > 4^th^ (Evans and Borsuk-Białynicka 2009: ch. 56).

57: Ilium contribution to acetabulum; 0 = 80 to 85 %; 1 = 60 to 65 % (Evans and Borsuk-Białynicka 2009: ch. 57).

58: Pubic flange; 0 = Absent; 1 = Present (Evans and Borsuk-Białynicka 2009: ch. 58).

59: Iliac blade; 0 = Long; 1 = Short (Evans and Borsuk-Białynicka 2009: ch. 59).

60: Anteriomedial portion of pubis; 0 = Not out-turned dorsally; 1 = Outturned dorsally (Evans and Borsuk-Białynicka 2009: ch. 60).

61: Dorsal edge of ilium; 0 = Horizontal; 1 = Steeply inclined (Evans and Borsuk-Białynicka 2009: ch. 61).

62: Pelvis; 0 = Solid plate; 1 = Small thyroid fenestra with broad pubic symphysis; 2 = Large thyroid fenestra with small pubic symphysis; 3 = Small thyroid fenestra does not extend to symphysis (Evans and Borsuk-Białynicka 2009: ch. 62).

63: Posterior border of the ischium; 0 = Generally smooth with perhaps a small posterorlaterally directed flange; 1 = Posterior flange; 2 = Distinct posterior process (Evans and Borsuk-Białynicka 2009: ch. 63; Apesteguía and Novas 2003: ch. 61).

64: Pelvic elements; 0 = Separate in adults; 1 = Fused or strongly held together in adults (Evans and Borsuk-Białynicka 2009: ch. 64).

65: Fibula-femur contact; 0 = Symmetrical condyles and contact; 1 = Asymmetrical, fibula sits in a recess on the lateral margin of distal end of the femur, asymmetrical contact (Evans and Borsuk-Białynicka 2009: ch. 65).

66: Fibula contact surface with astragalocalcaneum; 0 = Small part of distal end; 1 = Most of distal end (Evans and Borsuk-Białynicka 2009: ch. 66).

67: Astragalus-calcaneum; 0 = Separate elements throughout ontogeny; 1 = Fuses or at least strongly sutured prior to scapulocoracoid (Evans and Borsuk-Białynicka 2009: ch. 67).

68: Lateral centrale of pes; 0 = Discrete throughout ontogeny; 1 = Fused to astragalus prenatally (Evans and Borsuk-Białynicka 2009: ch. 68).

69: Distal tarsal 1; 0 = Present; 1 = Absent (Evans and Borsuk-Białynicka 2009: ch. 69).

70: Distal tarsal 2; 0 = Present; 1 = Absent (Evans and Borsuk-Białynicka 2009: ch. 70).

71: Distal tarsal 5; 0 = Present at some point during ontogeny; 1 = Absent or fused in embryo (possibly fused to the 5th metatarsal) (Evans and Borsuk-Białynicka 2009: ch. 71).

72: Metatarsal 5; 0 = Straight; 1 = Inflected, somewhat hooked but without enlarged plantar tubercles; 2 = Hooked and inflected, angulated and proximally and with enlarged medial and lateral plantar tubercles (Evans and Borsuk-Białynicka 2009: ch. 72).

73: Tibia bears distal ridge for contact with astragalus; 0 = Absent; 1 = Present (Evans and Borsuk-Białynicka 2009: ch. 73).

74: Contact between astragalocalcaneum and distal tarsal 4; 0 = No overlap or tongue in groove articulation; 1 = Process from distal tarsal 4 extends under astragalocalcaneum; 2 = Tongue in groove articulation (Evans and Borsuk-Białynicka 2009: ch. 74).

75: Premaxilla lateral process (modified); 0 = Premaxilla underlaps maxilla and does not exclude maxilla from nares; 1 = Premaxilla underlaps maxilla and dorsally expanded to exclude maxilla from nares; 2 = Overlaps maxilla but does not exclude maxilla from nares; 3 = Overlaps maxilla and excludes maxilla from nares (similar to Evans and Borsuk-Białynicka 2009: ch. 75; Apesteguía and Novas 2003: ch. 7; Sues et al. 1994: ch. 1; Reynoso, 1997: ch. 43 but modified from two states to four to describe the nature of the overlap involved).

76: Cervical vertebrae; 0 = Not elongate; 1 = Elongate (Evans and Borsuk-Białynicka 2009: ch. 76).

77: Astragalus and calcaneum; 0 = Without specialised joint; 1 = With specialised joint (Evans and Borsuk-Białynicka 2009: ch. 77).

78: Enlarged palatine tooth row; 0 = Absent; 1 = Present (Sues et al. 1994: ch. 26; Reynoso, 1997: ch. 35).

79: Articular surface of the lower jaw; 0 = Mesiodistally short (probably means orthal jaw movement); 1 = Mesiodistally long (propalinal movement possible) (reworded and recoded from Sues et al. 1994: ch. 34; Reynoso, 1997: ch. 19).

80: Premaxillary tooth structure; 0 = Discrete; 1 = Chisel-like (Apesteguía and Novas 2003: ch. 50; Reynoso 1996: ch. 33; Wu 1994: ch. 25; Sues et al. 1994: ch. 21).

81: 2nd sacral vertebra posterior edge; 0 = Process; 1 = Flange; 2 = No process (Apesteguía and Novas 2003: ch. 60; Reynoso 1996: ch. 40).

82: Number of pterygoid tooth rows on the anterior process of the pterygoid; 0 = Three or more rows; 1 = Two rows; 2 = One row or small patch; 3 = No teeth (Apesteguía and Novas 2003: ch. 56; Reynoso 1996: ch. 38).

83: A prominent skirt of secondary bone on the dentary; 0 = Absent; 1 = Present (new character but see Whiteside 1986; Jones 2006).

84: Parietal crest; 0 = Absent; 1 = Present (Apesteguía and Novas 2003: ch. 60; Reynoso 1996: ch. 40).

85: Obvious dental regionalisation into anterior and posterior sections; 0 = Absent; 1 = Present (Reynoso 1996: ch. 30).

86: Degree of tooth fusion (anterior marginal teeth); 0 = Clear boundary; 1 = Boundary between tooth and bone unclear; 2 = No obvious boundary between tooth and bone (new character; similar to Reynoso 1996: 27 but not the same).

87: Degree of tooth fusion (posterior marginal teeth); 0 = Clear boundary; 1 = Boundary between tooth and bone unclear; 2 = No obvious boundary between tooth and bone (new character; similar to Reynoso 1996: 26 but not the same).

88: Tooth replacement (anterior marginal teeth); 0 = Frequent (lots of replacement pits and tooth spaces); 1 = Slow (spaces and tooth replacement pits rare, tips may be worn); 2 = No evidence of tooth replacement, no spaces, teeth often clearly worn (new character; similar to Reynoso 1996: 27 but not the same).

89: Tooth replacement (posterior marginal teeth); 0 = Frequent (lots of replacement pits and tooth spaces); 1 = Slow (spaces and tooth replacement pits rare, tips may be worn); 2 = No evidence of tooth replacement, no spaces, teeth often clearly worn (new character; similar to Reynoso 1996: 27 but not the same).

90: Tooth location (posterior marginal teeth) (see also character 40); 0 = In shallow sockets or depressions; 1 = Against a low labial wall that is taller than any lingual wall; 2 = Against a very prominent labial wall (pleurodonty); 3 = On the crest of the jaw bone (acrodonty) (similar to Evans and Borsuk-Białynicka 2009: ch. 40; Reynoso 1996: ch. 26; Sues et al. 1994, ch19, Apesteguía and Novas 2003: ch. 43; but modified to include only to the anterior teeth and four states used rather than two or three to distinguish tooth location).

91: Lingual subdental shelf anteriorly; 0 = Absent; 1 = Present and at least as wide as the bases of the teeth (new character).

92: Posterior extension of the dentary; 0 = Absent; 1 = Reaches point level with the articular (reworded from Reynoso 1996: ch. 23; Sues et al. 1994: ch. 13).

93: Coronoid process of the dentary; 0 = Absent; 1 = Some expansion; 2 = Notable (extra state compared to Reynoso 1996: ch. 24; Sues et al. 1994: ch. 14; reworded from Apesteguía and Novas 2003: ch. 40 to account differences between early lepidosauromorphs).

94: Posterior flanges on the posterior maxillary teeth; 0 = Absent; 1 = Short; 2 = Long (Apesteguía and Novas 2003: ch. 51; Reynoso 1996: ch. 34).

95: Level of the jaw joint relative to the long axis of the tooth row; 0 = Level or above the level of the tooth row; 1 = Below the level of the tooth row (new character).

96: Scapulocoracoid; 0 = Coossified in mature adult; 1 = Separate elements in mature adult (Dupret 2004: ch. 96).

97: Anterior process of interclavicle; 0 = Prominent anterior process; 1 = Short process; 2 = No process (modified from Dupret 2004: chs 64, 65 and 66).

98: Vertebral number; 0 = 35 presacral vertebrae or less; 1 = more than 35 prescral vertebrae (modified from Dupret 2004: ch. 61).

99: Anterolabial flanges on the dentary teeth; 0 = Absent; 1 = Short; 2 = Long (Apesteguía and Novas 2003: ch. 57; Reynoso 1996: ch. 39).

100: Anteromedial flanges on the dentary teeth; 0 = Absent; 1 = Short (Apesteguía and Novas 2003: ch. 58).

Character coding

Coding was carried out using the references listed above as well as direct examination of material by either MEHJ or SEE. Abbreviations: A, 0&1; B, 0&1&2; C, 1&2&3; D, 2&3; E, 0&2; F, 1&2; G, 0&2&3; H, 0&3.

*Petrolacosaurus*

00000 00000 00000 00000 00100 00001 00000 000?0 ?0000 0000? ?0000 00000 1000? 00000 000?0 10000 00000 00000 00000 00000

*Youngina*

01000 00000 00000 00000 00000 01101 00000 00000 00010 00000 ?0000 00010 00100 ????? ?0?00 00000 10000 00000 00001 02000

*Czatkowiellia*

010?? ?1013 00000 00000 1??0? 100?1 0010? ???00 ?0??? 112?? ????? ????? 0???? ????? ????0 1?000 ?0000 00000 0??00 ???00

*Prolacerta*

000E1 11002 00000 00000 0A001 10001 00000 011?0 ?0001 1100? ?0010 01000 00?00 00100 12?02 110?0 20000 00000 00001 02?00

*Paliguana*

00000 0000? ???01 ?10?? ????? 1?0?0 ?0?0? ??1?? ????? ????? ????? ????? ????? ????? ????? ????? ???0? ????? ????? ?????

*Saurosternon*

????? ????? ????? ????? ????? ????? ????? ????? ?0001 1F00? 1010? 0???? ?000? ?0000 0001? 00??? ????? ????? ????? 020??

Kuehneosauridae

00021 11003 01-11 11100 0A000 01000 00000 00110 0?001 01211 ?0?1? 11000 1H00? 00??? ?100D 00000 00000 00000 00000 ??000

*Marmoretta*

1?02? ?0013 0??00 000?1 01??? 00010 01011 01?11 0000? 111?? ????? ?11?? ????? ????? ????0 0?000 ?0010 00001 00000 ???00

*Sophineta*

1?001 10113 0000? 010?? ????? 110?0 00?0? ????1 0100? 001?? ????? ????? ???0? ????? ????0 0?000 ?0000 11001 A000? ???00

Squamata

111B1 10A-G 10111 01111 11112 11010 AA2AA 11A1C A110A 201A1 21111 21101 12111 11111 13120 000A0 BD0AA BBBBC 10A00 ABAA0

*Gephyrosaurus*

11001 11112 01-00 01001 1110? 11000 01111 01112 01100 00000 ?010? ?1101 11111 ?11?? 12?20 00110 10001 01012 11100 02??0

*Diphydontosaurus*

21001 11112 01-00 01001 1110? 11000 00111 01112 ???0? ????? ????? ????? ????? ????? ????0 ??110 ?0001 02123 11100 ????0

Vellberg Jaw

????? 1???? ????? ????? ????? ????? ????? ????2 ????? ????? ????? ????? ????? ????? ????? ????? ??0?1 02123 1?1?? ????0

*Whitakersaurus*

????? ????? ????? ????? ????? ????? ????? ????2 ????? ????? ????? ????? ????? ????? ????? ????? ??0?1 12123 1???? ????0

*Rebbanasaurus*

????? ????? ????? ????? ????? ????? ????? ????3 ????? ????? ???? ????? ????? ????? ????? A??1?0 ??1?1 22223 0??1? ???11

*Planocephalosaurus*

21001 11112 01-01 01001 ?110? 11000 00?01 01113 ??100 100?? ?0100 ?1101 11111 ?1??? ?2??0 00110 20001 22223 11210 02?10

*Clevosaurus hudsoni*

21001 11111 00001 01001 ?110? 11000 00?01 01113 ??100 100?? ?0100 ?1101 11111 ????? ?2??1 00101 21101 22223 01220 02020

*Palaeopleurosaurus*

21001 11112 01-01 00011 ?110? 110?1 01?01 011?3 ??000 100?? ?0100 210?1 11211 01??? ?2??0 00101 23111 22223 01220 1212?

*Pleurosaurus*

21001 1-003 01-0? 0?0?1 ???0? 1?0?1 00?0? ??1?3 ??0?? ????? ?0100 1???? ?1211 ?1??0 11??A 00101 13111 22223 01110 12120

*Kallimodon*

????? ????? ????? ??011 ?1??? ????0 ?0??1 010?3 ?1100 ?00?? ?0000 11101 11211 01110 12??? 001?1 23111 22223 01221 12000

*Priosphenodon*

20001 11013 ?0001 0?011 ?11?? ?10?0 ?0?01 010?3 ????0 ????? ????? ????? ????? ????? ????1 ??111 23111 22223 01211 ???11

*Sphenodon*

21001 11110 01-01 00011 1110? 11000 00101 01013 ?1100 10000 20100 21101 11111 01110 12020 00111 03111 22223 01211 02011

**Fossil calibrations used in the main analysis**

We employ 14 calibration points for estimating the divergence time of Lepidosauria, crown Squamata and constituent clades. Parham et al. (2012) recently suggested five steps to evaluate how well justified a potential calibration point may be:

1) Museum numbers of specimen(s) that demonstrate all of the relevant characters and provenance data should be listed. Referrals of additional specimens to the focal taxon should be justified.

2) An apomorphy-based diagnosis of the specimen(s) or an explicit, up-to-date, phylogenetic analysis that includes the specimen(s) should be referenced.

3) Explicit statements on the reconciliation of morphological and molecular data sets should be given.

4) The locality and stratigraphic level (to the best of current knowledge) from which the calibrating fossil(s) was/were collected should be specified.

5) Reference to a published radioisotopic age and/or numeric time scale and details of numeric age selection should be given.

Within the limits of the current lepidosaur fossil record and geological knowledge we follow these steps to provide context for each calibration point.

As others have already discussed the greater the certainty that a fossil taxon represents a particular lineage (with a clear set of synapomorphies) the more likely it is to underestimate the actual age of a divergence indicated by those synapomorphies (e.g. Magallón 2004; Anderson 2007). However, to avoid implying that the ages of fossils are accurate to the nearest 100,000 yrs (Parham and Irmis 2008) we round down our dates to the nearest million years. Although rounding down increases the likelihood that the fossil underestimate the timing of divergence even further it does ensure that the minimum constraint represents the uppermost limit of the relevant time interval (Parham et al., 2012). Therefore, it allows the minimum age to be used as a hard minimum constraint.

CPX

Node: Archosauromorpha–Lepidosauromorpha node (Benton and Donoghue 2007) also previously referred to as the Crocodile–lizard split (Reisz and Muller 2004).

Fossil taxon: *Protorosaurus* sp.

Main reference: Evans and King 1993

Specimen number(s)/Material: TWCMS (Sunderland Museum, Tyne and Wear County Museums Service) S1348.1 (partial skeleton including cervical vertebrae, caudal vertebrae, parts of the pectoral girdle, partial squamosal and partial dentary with teeth)

Apomorphy based diagnosis: Oldest known archosauromorph. A recent phylogenetic analysis recovers *Protorosaurus* as the sister taxon of *Megalancosaurus* within Archosauromorpha (Gottmann-Quesada and Sander, 2009).

Reconciliation of morphology-molecular data set: n/a

Locality name: Quarrington Quarry (Evans and King 1993)

Paleobiology Data Base (PBDB) ID: 77197 (see also 81115, 84836, 85484)

Location: England, UK

Epoch: upmost Permian

Stage: Wuchiapingian

Stratigraphic level / horizon / unit: near the top of the Marl Slate (Pettigrew, 1979; Evans and King, 1993). Records of *Protorosaurus* from the Kupferschiefer of Germany are considered to be of similar age to those from the Marl Slate of northeast England (e.g. Sues and Munk, 1996; Gottmann-Quesada and Sander 2009). The Kupferschiefer is a thin bed (<4 m) of bituminous marl found in north-central Europe which was deposited under anoxic conditions in a stratified sea following a rapid marine transgression (Vaughan et al. 1989; García-Veigas et al, 2011). The Kupferschiefer corresponds to the base of the Zechstein cycles (PZ1 Werra: T1 in García-Veigas et al, 2011) and is overlain by the Zechstein Limestone (Ca1) (Vaughan et al. 1989; García-Veigas et al, 2011).

Radioisotopic age / numeric time scale information:

For *Protorosaurus*, Benton and Donoghue (2007) recommended a minimum age constraint of 259.7 Mya and a maximum constraint of 299.8 Mya. This was adoped by San Mauro (2010) who used a mean date of 279.75 Mya. The minimum suggested date was based on stratigraphy that suggests the Zechstein falls above the Illawarra Reversal, which is at the Wordian-Capitanian boundary, and fossils from the Zechstein I that suggest a Capitanian age (Gradstein et al. 2004). The maximum suggested date was based on *Apsisaurus* from the Asselian of USA which was considered to represent the oldest outgroup to Neodiapsida. However, *Apsisaurus* has recently been reinterpreted as a varanopid and thus nested within Synapsida (Reisz et al. 2010). The chronostratigraphic boundary of the Kupferschiefer also remains uncertain but sulphides provide an Re-Os isotopic date of 257.3±1.6 Mya (Brauns et al. 2003; Roscher and Schneider 2006; García-Veigas et al, 2011). More recently Benton et al. (2009) recommended a minimum of 255.9 Mya, and a maximum of 299.8 Mya. This was used by Mulcahy et al. (2012). Similarly, we use 255 Ma as a minimum age for *Protorosaurus*.

Date: 255 Mya.

CPY

Node: *Alligator-Passer montanus* (origin of Archosauria). This node has previously been referred to as the “bird-crocodile split” (e.g. Müller and Reisz 2005; Mulcahy et al. 2012), “bird-crocodile divergence” (Sanders and Lee 2007), or “crocodile-bird node” (e.g. Parham et al., 2012). A date at this node has been used in several analyses to constrain a significant branching point in the tree of amniotes (e.g. Hugall et al. 2007; Alfaro et al. 2009; Mulcahy et al. 2012). Müller and Reisz (2005) suggested 243 Mya as a minimum and 251 Mya as a maximum age for this divergence whereas Benton et al. (2009) proposed 239 Mya and 250.4 Mya respectively. However, recent work by Butler et al. (2011) and Parham et al. (2012) suggests alternative dates are worth considering.

Fossil taxon: *Ctenosauriscus koeneni*Main reference: Butler et al. 2011

Specimen number(s)/Material: GZG (Geowissenschaftliches Zentrum der Universität Göttingen, Göttingen, Germany) V.4191, holotype of *Ctenosauriscus koeneni* (Butler et al. 2011). Comprises a partial vertebral column (Butler et al. 2011).

Apomorphy based diagnosis: Oldest known certain archosaur. Several comprehensive morphological phylogenetic analyses show that *Ctenosauriscus* is part of a monophyletic grouping of poposauroid archosaurs (Butler et al. 2011). Characters supporting the placement of *Ctenosauriscus* within Ctenosauriscidae include elongation of the dorsal neural spines, and curvature of the neural spines along their length (Butler et al. 2011).

Reconciliation of morphology-molecular data set: n/a

Locality name: Bremketal (=‘‘Bremke dell’’).

Paleobiology Data Base (PBDB) ID: 109489

Location: Bremketal, east of Reinhausen, Germany
Epoch: Lower Triassic

Stage: latest Olenekian

Stratigraphic level / horizon / unit: The holotype of *Ctenosauriscus koeneni* was collected from the ‘‘Solling-Bausandstein’’ (upper Middle Buntsandstein: Solling Formation) at Bremke dell in Niedersachsen (Lower Saxony), northern Germany (Butler et al. 2011).

"stratigraphic work supports a well-constrained latest Olenekian age for the part of the upper Middle Buntsandstein" (Butler et al. 2011). The Olenekian-Anisian boundary is currently considered to lie at 247.2 Mya. Therefore, we conservatively use 247 Mya as a minimum bound.

Radioisotopic age / numeric time scale information: As discussed by Butler et al. (2011), radioisotopic data sets a date of 247.2 Mya for the Olenekian–Anisian boundary (Mundil et al. 2010) and examination of short eccentricity Milankovitch cycles within the Solling Formation provides a date of about 247.5 Mya for the base of the Solling (Kozur and Bachmann 2005, 2008). These two sources of information together suggests an approximate age of 247.5–247.2 Mya (latest Olenekian) for the holotype of *Ctenosauriscus koeneni* (Butler et al. 2011). Therefore, we use a conservative date of 247 Mya. Notes: There are several other crown group archosaurs known from Olenekian/Anisian boundary and show that Archosauria were widespread across the northern hemisphere at this time (Butler et al. 2011). For example, *Xilousuchus* from China (Wu 1981; Nesbitt 2011) and *Arizonasaurus babbitti* of USA (Nesbitt 2005, 2011). *Vytshegdosuchus* from the Yarenskian Gorizont unit of Russia (Tatarinov, 1960) is probably a little older but remains poorly known (Butler et al. 2011). Parham et al. (2012) proposed *Xilousuchus* *sapingensis* (Wu 1981) from the Triassic of China represented the best fossil taxon with which to calibrate this divergence. However, *Ctenosauriscus koeneni* is slightly older (247 vs 242 Mya) and it is at least as well analysed phylogenetically (Butler et al. 2011). Parham et al. (2012) also suggested 256 Mya for a conservative soft maximum bound based on *Archosaurus rossicus* from the late Permian of Russia (Sennikov and Golubev, 2006; Krassilov and Karasev 2009).

Date: 247 Mya (to 256 Mya).

CP1

Node: *Sphenodon*-*Varanus* (origin of Lepidosauria, the tuatara-lizard split)

Fossil taxon: the Vellberg jaw
Main reference: This paper

Specimen number(s)/Material: SMNS (Staatliches Museum für Naturkunde, Stuttgart, Germany) 91060 and 910601.

Apomorphy based diagnosis: See paper.

Reconciliation of morphology-molecular data set: n/a

Locality name: Vellberg locality

Paleobiology Data Base (PBDB) ID: n/a

Location: southern Germany
Epoch: Middle Triassic

Stage: Ladinian

Stratigraphic level / horizon / unit:

Both specimens were found in the same 50-100 mm thick mudstone layer at the top of the Untere Graue Mergel (lower grey marls) of the Lower Keuper (Erfurt Formation)

Radioisotopic age / numeric time scale information: Layer 6 of Schoch (2002) which has been dated to 240 Mya. Recent cyclostratigraphy indicates this bed is between 239 and 240 Mya. (Menning et al. 2005) which corresponds to the Ladinian part of the Middle Triassic (DSK 2005; Gradstein et al. 2012). Kozur and Bachman (2008) suggests a slightly earlier time period of 238-238.8 Mya for this unit based on zircon U-Pb dating. We therefore conservatively use the date of 238 Mya.

Notes: none.

Date: 238 Mya.

CP2

Node: *Eublepharis*–*Sphaerodactylus* (origin of Gekkonidae)

Fossil taxon: *Yantarogekko balticus*Main reference: Bauer et al. 2005

Specimen number(s)/Material: GAM (Deutsches Bernstein-Museum in Ribnitz-

Damgarten, Germany) 1400, holotype of *Yantarogekko balticus* (Bauer et al. 2005). Material comprises the anterior part of the body preserved in amber including the head, thorax and arms (Bauer et al. 2005).

Apomorphy based diagnosis: Oldest known member of Gekkonidae. Referal of *Yantarogekko balticus* to Gekkonidae is supported by a number of characters including the absence of moveable eyelids, and presence of enlarged, scansorial pads, straight, undivided scansorial plates along the length of the digits and a greatly reduced, but strongly clawed first digit (Bauer et al. 2005). However, there are no characters with which to infer a more specific relationship to extant genera within the Gekkonidae (Bauer et al. 2005).

Reconciliation of morphology-molecular data set: n/a

Locality name: Samland Peninsula

Paleobiology Data Base (PBDB) ID: n/a

Location: Kaliningrad District, Russia
Epoch: Lower Eocene

Stage: Lutetian?

Stratigraphic level / horizon / unit: Baltic Amber

Radioisotopic age / numeric time scale information: Radiometric ages for the marine strata that contains Baltic amber are 44.1±1.1 and 47.0±1.5 Mya, based on 40Ar/40K analyses of glauconite (Ritzkowski, 1997; Wolfe et al. 2009). Therefore, we conservatively use 44 Ma as a minimum bound.

Notes: Mulcahy et al. (2012) chose *Yantarogekko* to calibrate the most recent common ancestor of gekkotans but used a date of 54 Mya with little explanation.

Date: 44 Mya.

CP3

Node: *Xantusia*-*Cordylus*

Fossil taxon: *Palaeoxantusia fera*Main reference: Sullivan 1991

Specimen number(s)/Material: AMNH (American Museum of Natural History, USA) 2678, referred specimen (Sullivan 1991). Comprises a partial dentary (Sullivan 1991).

Apomorphy based diagnosis: Possible stem xantusiid. The diagnosis and phylogenetic relationships of this taxon are in need of revision (Smith 2009b). Nevetheless, material of a similar morphotype is known from several localities in large sample sizes (Hecht 1956; Estes 1976; Sullivan 1982; 1991; Schatzinger 1980; Smith 2009b). Potential characters linking this taxon to xantusiids include a closed Meckelian canal, splenial fused or partly fused to the dentary, a coronoid process on the dentary, and deep nasal process of the premaxilla (Estes 1976; Smith 2009b).

Affinity: Earliest xantusiid-like lizard.

Notes: Material similar to *Palaeoxantusia fera* has also been described from the Palaeocene of Montana (Ekalaka Member, Medicine Rocks [‘previously the River Tongue Formation]; Estes 1976; Belt et al. 2002) and Wyoming (Fort Union Formation, Sullivan 1982), and also the Eocene of California (Mission Valley Fm, Schatzinger 1980) and Wyoming (lowermost Willwood Formation, Smith 2009).

Reconciliation of morphology-molecular data set: n/a

Locality name: Gidley and Silberling quarries

Paleobiology Data Base (PBDB) ID: 14906 (=Gidley Quarry)

Location: Crazy Mountains, Montana, USA
Epoch: Early-Late Palaeocene

Stage: lowest part of the Selandian

Stratigraphic level / horizon/ unit: Fort Union Formation.

Radioisotopic age / numeric time scale information: Gidley and Silberling quarries are considered to be "middle Torrejonian” and part of the “To2" zone (Lofgren et al. 2004, 2005). The rocks of Silberling quarry are of reversed polarity and are suspected to be correlated with magnetic polarity chron C27r (Butler et al. 1987). This chron was previously considered to span 63.1 Mya and 61.7 Mya (Gradstein and Ogg 2004; Ogg and Smith 2004; Peppe et al. 2009) but is now inferred to be 63.5 and 62.5 Mya (Gradsetin *et al*. 2012). We opt for the conservative value of 61 Ma as a minimum age.

Notes: Mulcahy et al. (2012) used a date of 65.2 Mya to constrain the divergence between cordylids and xantusiids based on *Palaeoxantusia fera* (Estes, 1976) and *Konkasaurus* (Krause et al. 2003).

Date: 61 Mya.

CP4

Node: Lacertidae-Amphisbaenia

Fossil taxon: *Plesiorhineura tsentasi*Main reference: Sullivan 1985

Specimen number(s)/Material: NP (University of New Mexico, USA) 596, holotype of *Plesiorhineura tsentasi* (Sullivan 1985). Material comprises a partial dentary (Sullivan 1985).

Apomorphy based diagnosis: Oldest known rhineurid amphisbaenian. Sullivan (1985) does not explicity state what characters of *Plesiorhineura tsentasi* support its identity as a rhineurid amphisbaenian but referral seems to be based mainly on tooth morphology and general features (Sullivan 1985).

Reconciliation of morphology-molecular data set: n/a

Locality name: Torreon Wash (BLM&UNM locality 77-1840),

Paleobiology Data Base (PBDB) ID: n/a

Location: Deer Mesa Quadrangle, New Mexico, USA
Epoch: Middle Paleocene?

Stage: ‘Torrejonian’? TO2

Stratigraphic level / horizon / unit: Upper part of the Nacimiento Formation (Sullivan 1985).

Radioisotopic age / numeric time scale information: Torreon Wash is considered to be "middle Torrejonian” and part of the “To2" zone (Lofgren et al. 2004, 2005). Other localities that are part of the “To2” zone are suspected to correlate with magnetic polarity chron C27r (Butler et al. 1987). This chron was previous considered to span 63.1 and 61.7 Mya (Gradstein and Ogg 2004; Ogg and Smith 2004; Peppe et al. 2009) but is now estimated to be 63.5 and 62.5 Mya (Gradstein *et al*. 2012). Correspondingly, correlations based on pollen from the Nacimiento Formation suggest the fossil bearing zones are at least 62 Mya (Williamson et al. 2008). Further work is in process which may refine estimates (Tremaine 2011). We therefore opt for the conservative value of 61 Mya as a minimum bound.

Notes: Wiens et al. (2006) and Mulcahy et al. (2012) constrain this node using the fossil taxon *Hodzhakulia* (Nessov 1985). However, this fossil taxon from the Cretaceous of Asia is based on a dentary that does not show any unambigious amphisbaenian chartacters (Kearney, 2003). Wiens et al. (2006) used *Plesiorhineura* to constrain the minimum age of origination for Rhineuridae but used a date of 60.5 Mya was rather than 61 Mya.

Date: 61 Mya.

CP5

Node: *Python*-*Elgaria* (Serpentes-Anguimorpha)

Fossil taxon: *Dorsetisaurus* sp.
Main reference: Prothero and Estes 1981

Specimen number(s)/Material: AMNH (American Museum of Natural History) 27646 and 27647, referred material (Prothero and Estes 1981). Material comprises partial dentaries.

Apomorphy based diagnosis: Oldest known certain anguimorph or stem-anguimorph (Conrad 2008). Phylogenetic analysis based on morphological data place *Dorsetisaurus purbeckensis* as a basal platynotan: sister taxon to *Varanus*, *Heloderma*, *Shinisaurus* and a number of fossil taxa including *Parasaniwa* and mosasaurs (Conrad 2008, Conrad et al. 2011a). A combined evidence analysis still recovers *Dorsetisaurus purbeckensis* as a nonanguiform anguimorph and thus much less deeply nested (Conrad 2011a). The reliability of this calibration point rests on whether the material referred to *Dorsetisaurus* sp. is indeed related to the material from the UK. Neverthless, the lanceolate teeth shared between different specimens do seem highly characteristic (Prothero and Estes 1981; Evans and Chure 1999).

Notes: Further material exists undescribed from other Morrison localities (e.g. the Brushy Basin Member and Fruita, Evans and Chure 1999) plus similar material is known from the Kimmeridgian of Guimarota, Portugal (Seiffert 1973; Broschinski, 2000). The type material of *Dorsetisaurus* from the Purbeck Limestone Formation (UK) includes jaws, skull elements and braincase (BMNH R.8129). The latter particularly supports assignment to Anguimorpha (Conrad 2008).

Reconciliation of morphology-molecular data set: n/a

Locality name: Quarry 9

Paleobiology Data Base (PBDB) ID: n/a

Location: Quarry 9, Como Bluff
Epoch: Late Jurassic

Stage: Kimmeridgian

Stratigraphic level / horizon / unit: Brushy Basin Member, Morrison Formation

Radioisotopic age / numeric time scale information: Isotopic dating using single crystal ^40^Ar/ ^39^Ar Laser-Fusion ages and bulk ^40^Ar/ ^39^Ar step-heating ages from the top and bottom of the Brushy Basin Member provides the dates of 148. ±0.5 and 150.3 ±0.3 Mya respectively (Kowalis et al. 1998). Therefore, we use the conservative value of 148 Mya as a minimum bound.

Date: 148 Mya.

CP6

Node: *Varanus*-*Lanthanotus*

Fossil taxon: *Saniwa ensidens*Main reference: Rieppel and Grande, 2007.

Specimen number(s)/Material: FMNH (Field Museum of Natural History, Chicago, USA) PR2378 (Rieppel and Grande, 2007). Comprises a nearly complete skeleton with some skin and cartilage preservation.

Apomorphy based diagnosis: A relationship between *Saniwa ensidens*–*Varanus* is supported by 13 unambiguous synapomorphies including a short splenial, an enlarged lacrimal foramen, the absence of contact between the jugal and prefrontal, the absence of an intermedium from the manus, and absence of pterygoid teeth (Conrad et al. 2011a).

Reconciliation of morphology-molecular data set: Detailed phylogenetic analyses using both morphological and combined morphological+molecular data sets (the latter involving 175 fossil and extant anguimorphs, and 2281 parsimony-informative characters [315 morphological characters and 1969 molecular characters]) recover *Saniwa* as the immediate sister taxon to *Varanus* (Conrad et al. 2011ab).

Locality name: Tynsky Quarry, Thompson Ranch, F-2 locality H of Grande and Bucheim (1994).

Paleobiology Data Base (PBDB) ID: 15853

Location: Lincoln County, southwestern Wyoming, USA
Epoch: early Eocene

Stage: Wasatachian (Ypresian)

Stratigraphic level / horizon/ unit: Fossil Butte member

Radioisotopic age / numeric time scale information: The Green River Formation lies entirely within Chron C23r and is overlain by the Wasatch Formation (Clyde et al. 2001). Moreover the upper boundary of the Chron C23r is considered to be 51.75 Ma. The age of the Eocene Green River Formation has been inferred using ^238^U-^206^Pb ages of zircon, ^40^Ar/^39^Ar to provide a minimum date of 48.5 Mya (Smith et al. 2010).

Notes: Trunk vertebrae described as perhaps comparable to *Saniwa* (Sullivan 1982) are also known from the Fort Union Formation (sometimes called the ‘Lebo Formation, which is dated to about 62 Mya) but these are not diagnostic. Similarly Smith (2009b) described material possibly relating to *Saniwa* from the Willwood Formation of the Bighorn Basin but this is very incomplete.

Date: 48 Mya.

CP7

Node: *Heloderma*-*Anniella*

Fossil taxon: *Primaderma nessovi*

Main reference: Nydam 2000

Specimen number(s)/Material: OMNH (Sam Noble Oklahoma Museum of

Natural History, Norman) 26742 (Nydam 2000). Material comprises a partial right maxilla.

Apomorphy based diagnosis: Oldest fossil taxon more closely related to *Heloderma* than any other living taxon. The initial phylogenetic analysis based entirely on morphological characters placed *Primaderma nessovi* in a clade with the fossil taxa *Gobiderma,* *Paraderma*, *Primaderma*, *Estesia*, *Eurheloderma*, *Lowesaurus*, and the extant *Heloderma* (Nydam 2000). Subsequent analyses have recoved a similar results (e.g. Conrad 2008) including a combined morphological and molecular analysis (175 fossil and extant anguimorphs, and 2281 parsimony-informative characters [315 morphological characters and 1969 molecular characters]) by Conrad et al. (2011a, see the phylogenetic tree in Fig. 20).

Notes:

Other known monstersaurs such as *Gobiderma* and *Estesia* are Late Cretaceous in age but are diverse and found in both North America and east Asia (Conrad 2011b). Note that *Primaderma nessov* is not the sister taxon to all other monstersaurs and is more deeply nested than two Late Cretaceous taxa (*Parasaniwa* and *Parviderma*).

Reconciliation of morphology-molecular data sets: The precise placement of Monstersauria remains problematic. The results of our analysis match those of Townsend et al. (2004) with *Heloderma* as the sister taxon to *Anniella*+Anguidae. The molecular analysis of Wiens et al. (2010) differs slightly with *Xenosaurus* nesting within this grouping rather than just outside it. By contrast some analyses based on morphological data recover Monstersauria as the sister taxon to Varaniodea in a group referred to as Platynota (Conrad 2008; Wiens 2010). Associated with this problem is some confusion over the precise content or definition of Anguioidea. Conrad et al. (2011a, summarised in table 1, 254) provided a node based definition of Anguioidea as “*Anguis fragilis* + *Anniella pulchra* + *Xenosaurus grandis*”. Thus, if Monstersauria is the sister taxon to *Anniella*+Anguidae exclusive to *Xenosaurus grandis* it is included within Anguioidea (Conrad 2011b). Also it means Anguimorpha comprises the major clades of Anguioidea and Goannasauria (Conrad et al. 2011a, 269). Alternatively, if Monstersauria nest outside *Anguis fragilis* + *Anniella pulchra* + *Xenosaurus grandis* under the same node based definition of Conrad et al. (2011a, summarised in table 1, 254) it means Anguioidea and Monstersauria are sister taxa (Anguioidea of Conrad 2011a: 268, table 1 and Conrad 2011b: 67, 69, figure 56). Using the Anguioidea of Camp (1923) Monstersauria would be included regardless as to whether or not it was the sister taxon or nested within *Anguis fragilis* + *Anniella pulchra* + *Xenosaurus grandis*.

Locality name: OMNH locality V695

Paleobiology Data Base (PBDB) ID: 14396

Location: Emery County, Utah, USA

Epoch: Cretaceous

Stage: Albian Cenomanian

Stratigraphic level / horizon / unit: upper part of the Mussentuchit Member of the Cedar Mountain Formation

Radioisotopic age / numeric time scale information: Multiple, consistent ^40^Ar/

^39^Ar measurements taken from volcanic ash associated with the fauna provide an average age of 98.39±0.07 Mya (Cifelli et al. 1997). Therefore, we opt for the conservative value of 98 Ma as a minimum bound. The extensive phylogenetic analysis of this specimen and detailed dating of its source unit make this record one the best potential calibrations for lepidosaurs.

This calibration point was been used by Wiens et al. (2006) although 99 Ma was used as a date rather than 98 Mya.

Date: 98 Mya.

CP8

Node: *Elgaria*-*Ophisaurus*

Fossil taxon: *Ophisaurus* sp.
Main reference: Klembara and Green 2010

Specimen number(s)/Material: IWCMS (Isle of Wight County Museum Service) 2006.35, referred specimen (Klembara and Green 2010). Material comprises a partial parietal.

Apomorphy based diagnosis: *Ophisaurus*-like fossil, oldest anguine. The absence of a crista postfovealis and presence of a broad and arch-like parietal notch supports referral to *Ophisaurus* (Klembara and Green 2010).

Reconciliation of morphology-molecular data set: n/a

Locality name: Newtown Bone Bed

Paleobiology Data Base (PBDB) ID: n/a

Location: Isle of Wight, UK
Epoch: Upper Eocene (Priabonian)

Stage: ‘Priabonian’

Stratigraphic level / horizon / unit: Osborne Member, Headon Hill Formation

Radioisotopic age / numeric time scale information: The Osborne member is overlain by the Bembridge Marls member and the base of this unit is thought to coincide with Chron 13n Hooker et al. 2009) which is estimated to begin about 33.6 Mya (Gradstein and Ogg 2004; Gale et al. 2006). The Osborne member probably lies within Chron 13r and is thus has an age of about 34 Ma (Gradstein and Ogg 2004; Hooker et al. 2009; Gradstein et al. 2012). Therefore we suggest a conservative age of 33 Ma for this record.

Notes: Two other anguines are known from the Eocene (*Headonhillia parva*) described in Klembara and Green (2010) and *Ophisauriscus quadrupes* from the Middle Eocene of Germany (Klembara 1981; Sullivan et al. 1999).

Date: 33 Mya.

CP9

Node: *Chamaeleo*+*Calumma*

Fossil taxon: *Chamaeleo* sp.
Main reference: Čerňanský 2010

Specimen number(s)/Material: SGDB (geological collection of the Bílina opencast mine at Merkur-North, Czech Republic (Bílina city) Ah-772, referred to *Chamaeleo* sp. (Čerňanský 2010). Material comprises a partial right dentary but it is associated with several other partial dentaries also referable to *Chamaeleo* sp. (e.g. 845 SGDB, 857 SGDB, 859 SGDB, 860 SGDB, Čerňanský 2010).

Apomorphy based diagnosis: Referred to *Chamaeleo* sp. because of the following features which are identical to those in *Chamaeleo chamaeleon*: a distinct angular process, and deep surangular sinus between the surangular process and the coronoid process of the dentary (Čerňanský 2010).

Reconciliation of morphology-molecular data set: n/a.

Locality name: Merkur-North locality, the Czech Republic

Paleobiology Data Base (PBDB) ID: n/a

Location: opencast mine near Chomutov, the Czech Republic
Epoch: lower Miocene

Stage: Eggenburgian

Stratigraphic level / horizon / unit: The fossil material derives from the grey calcareous marls at the base of the “Main Brown Coal Seam” (reworked volcanic ash) (Čerňanský 2010).

Radioisotopic age / numeric time scale information: The Merkur-North locality is considered to represent the MN3 which according to various stratigraphic correlations spans about 19 Mya and 20.5 Mya (Steininger and Wessely 2000). Therefore, we use the conservative date of 19 Mya as a calibration point.

Notes:

Similar material (cf. *Chamaeleo* sp.) is known from the Wintershof-West locality of Germany which exposes the Eggenburg unit (Moody and Roček 1980). Although Moody and Rocek (1980) reported a date of 26 Mya, more recent estimates (Steininger and Wessely 2000) placee it at about 19 Ma. Further chaemaeleon-like material with a similar morphology to chamaeleons has been described from slightly younger beds of the same region Čerňanský (2010).

Date: 19 Mya.

CP10

Node: *Physignathus*-*Ctenophorus*

Fossil taxon: *Physignathus* sp.
Main reference: Covacevich et al. 1990

Specimen number(s)/Material: QM F18004 and QM F18007 (both partial dentaries)

Apomorphy based diagnosis: Referable to *Physignathus* sp. according to direct comparisons with modern material (Covacevich et al. 1990).

Reconciliation of morphology-molecular data set: n/a

Locality name: Camel Sputum Site, Riversleigh

Paleobiology Data Base (PBDB) ID: n/a

Location: Australia
Epoch: early Miocene

Stage: ?unknown

Stratigraphic level / horizon / unit: Faunal Zone B (Archer et al. 1989, 2006; Travouillon et al. 2009). Material is known from the following sites: Camel Sputum, Inabeyance, Hiatus Site, Upper Site, Neville's Garden, RSO, Wayne's Wok (Archer et al. 2006).

Radioisotopic age / numeric time scale information: Camel Sputum Site, Riversleigh

Is part of Faunal Zone B (“System B”) which lies between Faunal Zone A and C. System A sites share mammalian species with central Australian deposits [Etadunna Fm] dated magnetostratigraphically to Late Oligocene (~24–25 Mya, Woodburne et al., 1993); System C contains a sequence of assemblages that probably span the Middle Miocene (~16.3 to 10.4MYA); and System B is intermediate in age, probably Early Miocene (Archer et al., 1997)." Hence, System B = between approximately 16 and 24 Mya. Therefore we use a conservative date of 16 Mya.

Notes: Material referable to *Physignathus* sp. also known from the contemporaneous Wayne's Wok site of Riversleigh (Covacevich et al. 1990).

Date: 16 Mya.

CP11

Node: *Gambelia*-*Anolis*

Fossil taxon: *Afairiguana avius*Main reference: Conrad et al. 2007

Specimen number(s)/Material: FMNH (Field Museum of Natural History, Chicago, USA) PR 2379, holotype of *Afairiguana avius* (Conrad et al. 2007). Material comprises nearly a complete skeleton preserved in ventral view (Conrad et al. 2007).

Apomorphy based diagnosis: the oldest *Anole*-like lizard and the earliest pleurodontan iguanian (Conrad et al. 2007). Phylogenetic analysis of 50 taxa with 202 morphological characters places *Afairiguana avius* within Iguania and Polychrotidae (Conrad et al. 2007). A clade containing *Polychrus*, Leiosaurinae, Anisolepinae and *Afairiguana avius* is supported by the following characters: absence of a jugal-squamosal contact, a reduced crista prootica which does not extend ventrolaterally, placement of the anterior inferior alveolar foramen completely within the splenial (no dentary contribution to the border). A further character, proximally expanded clavicles that are notched/fenestrated, supports a less inclusive (and unresolved) grouping of Leiosaurinae + Anisolepinae + *Afairiguana avius* (Conrad et al. 2007).

Notes: The next oldest pleurodontan iguanian known is *Geiseltaliellus longicaudus* which is considered to be from within Corytophanidae (Rossmann, 1999, 2000; Smith 2009a).

Reconciliation of morphology-molecular data set: n/a

Locality name: Warfield Springs Locality, Green River (Conrad et al. 2007). Locality K in Grande and Buchheim (1994).

Paleobiology Data Base (PBDB) ID: 85026

Location: Wyoming, USA.
Epoch: lower Eocene

Stage: Wasatachian? (Ypresian?)

Stratigraphic level / horizon / unit: Fossil Butte Member of the Green River Formation (Conrad et al. 2007). The Warfield Springs locality is not as well known as the F-1 and more northern F-2 localities of Grande and Buchheim (1994) but is considered to be age equivalent to the latter (Conrad et al. 2007). Radioisotophic age / numeric time scale information: The Green River Formation lies entirely within Chron C23r and is overlain by the Wasatch Formation (Clyde et al. 2001). Moreover the upper boundary of the Chron C23r is considered to be 51.75 Mya. The age of the Eocene Green River Formation has been inferred using ^238^U-^206^Pb ages of zircon, ^40^Ar/^39^Ar to provide a minimum date of 48.5 Mya (Smith et al. 2008; 2010).

Date: 48 Mya.

CP12

Node: *Shinisaurus*-*Elgaria*

Fossil taxon: *Dalinghosaurus longidigitus*Main reference: Evans and Wang 2005

Specimen number(s)/Material: GMV (Geological Museum of China, Beijing, China) 2127, referred specimen (Evans and Wang 2005). Comprises the posterior part of the body (tail, pelvis, and hind limbs) but see also IVPP V13282 (articulated skull). Many fully articulated skeletons available (Evans and Wang 2005).

Apomorphy based diagnosis:

An extinct taxon more closely related to *Shinisaurus* than any other living squamate. In a recent large combined phylogenetic analyses *Dalinghosaurus* was recovered as the sister taxon to *Shinisaurus* (Conrad et al. 2011a). Characters supporting this result include a frontal with a single anterior wedge when articulated and seen in dorsal view; an anterolateral prefrontal tuberosity; anterior flange of the prefrontal reaching the posterodorsal margin of the external naris; a small crista prootica, ventral downgrowths not defining a deep jugal fossa; a short tympanic crest on the quadrate; three bilateral premaxillary tooth positions (Conrad et al. 2011a).

Reconciliation of morphology-molecular data set:

Locality name: Sihetun Village, China.

Paleobiology Data Base (PBDB) ID: n/a

Location: near Beipiao City, western Liaoning Province, China
Epoch: Early Cretaceous

Stage: Barremian?

Stratigraphic level / horizon / unit: Yixian Formation Jianshangou Bed

Radioisotopic age / numeric time scale information: Details can be found in Wang and Zhou (2003).

Date: 128 Mya.

**How our fossil calibrations differ from Mulcahy et al. 2012**

A recent analysis of divergences within Lepidosauria used a set of calibration points that differ slightly from the ones used in our analysis. This warrants some specific discussion.

1. Mulcahy et al. (2012: node 1) use a minimum and maximum constraint of 312.3 Mya and 330.4 Mya respectively for the crown of Amniota: the “bird-mammal” split. These dates follow the reconmendations of Benton and Donoghue (2006). We do not use a constraint at this node.
2. For crown Diapsida, Mulcahy et al. (2012: node 2) follow Benton et al. (2009) using 255.9 Mya as a minimum and 299.8 Mya as a maximum age for this node. We use 255 Mya as a minimum constraint only (Node CPX).
3. Mulcahy et al. (2012: node 3) use the Carnian rhynchocephalian material of Sues and Olsen (1990) to provide a minimum date of 222.8 Mya for the crown of Lepidosauria (tuatara-lizard split). Revised stratigraphic work suggests this material is likely to be older than 228 Mya (**Sues and Hopson 2010;** Gradstein et al. 2012). Moreover, our analysis uses a new rhynchocephalian fossil from even older deposits and allow us to constrain this node to 238 Ma (Node CP1).
4. Mulcahy et al. (2012: node 4) follow Benton et al. (2009) using 239 Mya as a minimum and 250.4 Mya as a maximum age for the “bird-crocodile split”. We instead use 247 Mya and 256 Mya based on more recent work by Butler et al. (2011) and Parham et al. (2012) (Node CPY).
5. Mulcahy et al. (2012: node 5) use *Yantarogekko* to constrain the most recent common ancestor of gekkotans to 54 Mya. We employ *Yantarogekko* to constrain the divergence between *Eublepharis* and *Sphaerodactylus* (origin of Gekkonidae) but use the more recent date of 44 Mya based on radiometric dates from the marine strata that contains Baltic amber (Ritzkowski, 1997; Wolfe et al. 2009) (Node CP2).
6. Mulcahy et al. (2012: node 6) used *Palaeoxantusia fera* (Estes, 1976) and *Konkasaurus* (Krause et al. 2003) to provide a minimum date of 65.2 Mya for the divergence between xantusiids and cordylids. We also use *Palaeoxantusia fera* to constrain this node but opt for a slightly more conservative date of 61 Mya (Node CP3).
7. Mulcahy et al. (2012: node 7) use a number of Late Cretaceous fossils such as *Meniscognathus* (Estes, 1964) to give a minimum date of 70 Mya for the divergence between Teiidae and Gymnophthalmidae based . We do not constrain this node.
8. Mulcahy et al. (2012: node 8) used *Hodzhakulia* (Nessov, 1985) to constrain the divergence between amphisbaenians and Lacertidae to a minimum of 111 Mya. However, this fossil taxon is based on a partical dentary that does not show any unambigious amphisbaenian characters (Kearney, 2003). As argued by Hipsley et al. (2009) *Hodzhakulia* is problematic as a calibration point. We consider the fossil taxon *Plesiorhineura tsentasi* (Sullivan 1985), also used as a calibration fossil by Hipsley et al. (2009), to be more certainly amphisbaenian. This provides a more conservative divergence date of 61 Mya (CP4).
9. Mulcahy et al. (2012: node 9) used isolated vertebrae of *Coniophis* from the Lance Formation of Wyoming, USA (Marsh 1892; Estes, 1976) to constrain the origin of Alethinophidea to a minimum of 92.7 Mya. However, more recent work on this taxon that also included referred cranial material places *Coniophis* as an ancient stem-snake outside both Alethinophidea and Scolecophidia (Longrich et al. 2012). We do not constrain any node within Serpentes but we do use *Dorsetisaurus* from the Late Jurassic of North America (Prothero and Estes 1981; Conrad 2011a) to provide a minimum age for the divergence between *Python* and *Elgaria* (CP5).
10. Mulcahy et al. (2012: node 10) use several fossil taxa from the Cretacous of North America and Asia such as *Palaeosaniwa* (Bryant, 1989) to calibrate the *Shinisaurus*-*Varanus* divergence to a minimum of 70 Mya. We instead use *Dalinghosaurus* from the Early Cretaceous of China (Evans and Wang 2005) to constrain the divergence between *Shinisaurus* and *Elgaria* to 128 Mya (Node CP12) and *Saniwa* (Rieppel and Grande, 2007) to constrain the divergence between *Varanus* and *Lanthanotus* (Node CP6).
11. Mulcahy et al. (2012: node 11) use *Odaxosaurus* from the Late Cretaceous of North America (Bryant, 1989) to constrain the divergence between *Elgaria* and *Xenosaurus* to 70.6 Mya. As mentioned above we instead use *Dalinghosaurus* from the Early Cretaceous of China (Evans and Wang 2005) to constrain the divergence between *Shinisaurus* and *Elgaria* (Node CP12). We also use material referred to *Ophisaurus* from the Eocene of the UK (Klembara and Green 2010) to provide a minimum age for the divergence between *Ophisaurus* and *Elgaria* (Node CP8).
12. Mulcahy et al. (2012: node 12) use various fossils from the Late Cretaceous of Asia (Gao and Norrell, 2000) to constrain the minimum age of Iguania to 70 Mya. We do not constrain this node but we do use three constraints within Iguania. The first uses fossil material referred to *Chamaeleo* (Čerňanský 2010) to constrain the divergence of *Chamaeleo* and *Calumma* to 19 Mya (CP9). The second uses *Afairiguana* from the Eocene of USA (Conrad et al. 2007) to constrain the divergence between *Gambelia* and *Anolis* to 48 Mya (CP11). The third uses *Physignathus* fossils from the Miocene of Australia (Covacevich et al. 1990) to constrain the divergence between *Physignathus* and *Ctenophorus* to a minimum of 16 Mya (CP10).
13. Mulcahy et al. (2012: node 13) use *Primaderma* from the Late Cretaceous of North America (Nydam 2000) to constrain the most recent common ancestor of helodermatids, anguids, and xenosaurids to a minimum date of 99.6 Mya. This calibration was also used by Vidal and Hedges (2005) and Wiens et al. (2006). Similarly we use *Primaderma* to constrain the divergence between *Heloderma* and *Anniella* to a minimum of 98 Mya (CP7).
14. Mulcahy et al. (2012: node 14) used various fossil taxa such as *Sauriscus* and *Contogenys* from the Late Cretaceous (Estes 1964, 1969) to constrain the divergence of crown group scincids to a minimum of 70 Mya. We do not constrain this node.

**SM Figures**

**B**

**C**

**D**

**A**

Figure S1.1. Partial rhynchocephalian dentaries from the Vellberg locality of Germany. A. Photo of SMS 91060. B. CT model of SMS 91060. C. Photo of SMS 91061. D. CT model of SMS 91060.


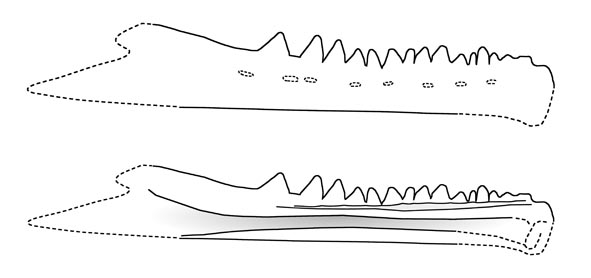


Figure S1.2. Tentative reconstruction of the Vellberg taxon based on SMS 91060 and Dentary SMS 91061. Approximate length = 15 mm.

**SM References**

Albert EM, San Mauro D, García-París M, Rüber L, Zardoya R: **Effect of taxon sampling on recovering the phylogeny of squamate reptiles based on complete mitochondrial genome and nuclear gene sequence data.** *Gene* 2009, **441:**12-21.

Alfaro ME, Santinia F, Brock C, Alamillo H, Dornburg A, Rabosky DL, Carnevale G, Harmon LJ: **Nine exceptional radiations plus high turnover explain species diversity in jawed vertebrates.** *Proc Nat Acad Sci* 2009, **106:**13420-13414.

Anderson CL: Dating divergence times in phylogenies. PhD thesis, Digital Comprehensive Summaries of Uppsala Dissertations from the Faculty of Science and Technology Acta Universitatis Upsaliensis, 2007.

Apesteguía S, Novas FE: Large Cretaceous sphenodontian from Patagonia provides insights into lepidosaur evolution in Gondwana. Nature 2003, 425:609-612.

Archer M, Godthelp H, Hand SJ, Megirian D: **Fossil mammals of Riversleigh, northwestern Queensland: preliminary overview of biostratigraphy, correlation and environmental change.** *Aust Zool* 1989, **25:**29-65.

Archer M, Hand SJ, Godthelp H, Creaser P: **Correlation of the Cainozoic sediments of the Riversleigh World Heritage fossil property, Queensland, Australia.** *Mémoires et Travaux de l’Institut de Montpellier de l’Ecole Pratique des Hautes Etudes* 1997, **21:**131-152.

Archer M, Derrick AA, Bassarova M, Beck RMD, Black K, Boles WE, Brewer P, Cooke BN, Crosby K, Gillespie A, Godthelp H, Hand SJ, Kear BP, Louys J, Morrell A, Muirhead J, Roberts KK, Scanlon JD, Travouillon KJ, Wroe S: **Current status of species-level representation in faunas from selected fossil localities in the Riversleigh World Heritage Area, northwestern Queensland.** *Alcheringa* 2006, **301:**1-17.

Bauer AM, Bohme W, Weitschat W: **An Early Eocene gecko from Baltic amber and its implications for the evolution of gecko adhesion.** *J Zool* 2005, **265:**327–332.

Belt ES, Diemer JA, Vuke SM, Beutner EC, Cole BS: **The Ekalaka Member of the Fort Union Formation, Southeastern Montana: designating a new member and making a case for estuarine deposition and bounding unconformities.** *Mont Bur Mines Geol Open-File Rep* 2002, 461:1–56

Benton MJ: **Classification and phylogeny of the diapsid reptiles.** *Zool J Linn Soc* 1985 **84:**97–164.

Benton MJ: **Phylogeny of the major tetrapod groups: morphological data and divergence dates.** *J Mol Evol* 1990, **30:**409-424

Benton MJ: *The Fossil Record 2*. New York: Chapman & Hall; 1993.

Benton MJ, Donoghue PCJ: **Paleontological evidence to date the tree of life.** *Mol Biol Evol* 2007, **24:**26-53.

Benton MJ, Donoghue PCJ, Asher RJ: **Calibrating and constraining the molecular clock.** In *The Timetree of Life*. Edited by Hedges SB, Kumar S. Oxford: Oxford University Press; 2009:35-86.

Borsuk−Białynicka M, Evans SE: **A long−necked archosauromorph from the Early Triassic of Poland.** *Palaeontologia Polonica* 2009, **65:**203-234.

Brauns CM, Patzold T, Haack U: **A Re-Os study bearing on the age of the Kupferschiefer black shale at Sangerhausen (Germany).** *XVth International Congress on Carboniferous and Permian Stratigraphy*. Utrecht, 2003, Abstracts, 66.

Bremer K: **Branch support and tree stability.** *Cladistics* 1994, **10:**295-304.

Broschinski A: **The lizards from the Guimarota mine.** In *Guimarota: a Jurassic Ecosystem*. Eidted by Martin T, Krebs B. Munich: Dr Friedrich Pfeil; 2000:59-68.

Broom R: **On the skull of a true lizard (*Paliguana whitei*) from the Triassic beds of South Africa.** *Rec Albany Mus* 1903, **1:**1-3.

Bryant LJ: **Non-dinosaurian lower vertebrates across the Cretaceous-Tertiary boundary in northeastern Montana.** *Univ Calif Publ Geol Sci* 1989, **134**:1-107.

Butler RF, Krause DW, Gingerich PD: **Magnetic polarity stratigraphy and biostratigraphy of Middle-Late Paleocene continental deposits of South-Central Montana.** J Geol 1987, **95:**647-657.

Butler RJ, Brusatte SL, Reich M, Nesbitt SJ, Schoch RR, Hornung JJ: **The sail-backed reptile *Ctenosauriscus* from the Latest Early Triassic of Germany and the timing and biogeography of the Early Archosaur radiation.** *PLoS ONE* 2011, e25693.

Camp CL: **Classification of the lizards.** *Bull Am Mus Nat Hist* 1923, **48:**289-481.

Camp CL: ***Prolacerta* and the protorosaurian reptiles.** *Part I. Am J Sci* 1945a, **243:**17–32.

Camp CL: ***Prolacerta* and the protorosaurian reptiles.** *Part II. Am J Sci* 1945b, **243:**84–101.

Carroll RL: **Permo-Triassic ‘lizards’ from the Karroo.** *Palaeontol Afr* 1975, **18:**71-87.

Carroll RL: **The origin of lizards.** In *Problems in Vertebrate Evolution*. Edited by Andrews SM, Miles RS, Walker AD. London: Linnean Society Symposium Series 4, Academic Press; 1977:359-396.

Carroll RL: **A pleurosaur from the lower Jurassic and the taxonomic position of the Sphenodontida.** *Palaeontol Abt A* 1985:189:1-28.

Carroll RL: *Vertebrate paleontology and evolution.* New York: W.H. Freeman; 1988.

Carroll RL, Wild R: **Marine members of the Sphenodontia.** In *In the shadow of the dinosaurs: Early Mesozoic tetrapods*. Edited by Fraser NC, Sues H-D. Cambridge: Cambridge University Press; 1994:70-83.

Čerňanský A: **A revision of chamaeleonids from the Lower Miocene of the Czech Republic with description of a new species of *Chamaeleo* (Squamata, Chamaeleonidae).** *Geobios* 2010, **43:**605-613.

Cifelli RL, Kirkland JI, Weil A, Deino AL, Kowallis BJ: **High precision 40Ar/39Ar geochronology of North America's Late Cretaceous terrestrial fauna.** PNAS 1997, **94:**11163-11167.

Clyde WC, Sheldon ND, Koch PL, Gunnell GF, Bartels WS: **Linking the Wasatchian/Bridgerian boundary to the Cenozoic Global Climate Optimum: new magnetostratigraphic and isotopic results from South Pass, Wyoming.** *Palaeogeog Palaeoclim Palaeoecol* 2001, 167:175–199.

Colbert EH: ***Icarosaurus*, a gliding reptile from the Triassic of New Jersey.** *Am Mus Novit* 1966, **2246:**1-23.

Colbert EH: **The Triassic gliding reptile *Icarosaurus*.** *Bull Am Mus Nat Hist* 1970, **143:**85-142.

Cocude-Michel M: **Les rhynchocéphales et les sauriens des calcaires lithographiques (Jurassique supérieur) d’Europe Occidentale.** *Nouv Arch Mus Hist Nat Lyon* 1963, 7:1-187.

Conrad JL: **Phylogeny and systematics of Squamata (Reptilia) based on morphology.** *Bull Amer Mus Nat Hist* 2008, **310:**1-182.

Conrad JL, Ast JC, Montanari S, Norell MA: **A combined evidence phylogenetic analysis of Anguimorpha (Reptilia: Squamata).** *Cladistics* 2011a, **27:**230-277.

Conrad JL, Rieppel O, Gauthier JA, Norell MA: **Osteology of *Gobiderma pulchrium* (Monstersauria, Lepidosauria, Reptilia).** *Bull Amer Mus Nat Hist* 2011b, **362:**1-88.

Conrad JL, Reippel O, Grande L: **Re-assessment of varanid evolution based on new data from *Saniwa ensidens* Leidy, 1870 (Squamata, Reptilia).** *Novitates* 2008, **3630:**1-15.

Conrad JL, Rieppel O, Grande L: **A Green River (Eocene) polychrotid (Squamata: Reptilia) and a re-examination of iguanian systematics.** *J Paleontol* 2007, **81:**1365-1373.

Covacevich J, Couper P, Molnar RE, Witten G, Young W: **Miocene dragons from Riversleigh: new data on the history of the family Agamidae (Reptilia: Squamata) in Australia.** *Mem Queensland Mus* 1990, **29:**339-360.

Crawford NG, Faircloth BC, McCormack JE, Brumfield RT, Winker K, Glenn TC: **More than 1000 ultraconserved elements provide evidence that turtles are the sister group of archosaurs.** Biol Lett 2012 DOI:10.1098/rsbl.2012.0331

deBraga M, Rieppel O: **Turtles as diapsid reptiles***.* *Nature* 1996, **384:**453-455.

deBraga M, Rieppel O: **Reptile phylogeny and the interrelationships of turtles.** *Zool J Linn Soc* 1997, **120:**281-354.

DSK: Deutsche Stratigraphische Kommission (Hrsg.): **Stratigraphie von Deutschland IV – Keuper.** *Courier Forschungsinstitut Senckenberg*. 2005, 253: 296 S., 64 Abb., 50 Tab., 2 Taf.; Frankfurt am Main.

Dupret V: **The pleurosaurs: anatomy and phylogeny.** Rev Paléobiol 2004, **9:**61-80.

Estes R: **Fossil vertebrates from the Late Cretaceous Lance Formation, eastern Wyoming.** *Univ Calif Pub Geol Sci* 1964, **49:** 1-180.

Estes R: **A scincoid lizard from the Cretaceous and Paleocene of Montana.** *Breviora* 1969, **331:**1-9.

Estes R: **Middle Paleocene Lower vertebrates from the Tongue River Formation, Southeastern Montana.** *J Paleontol* 1976, **50:**500-520.

**Evans SE: The skull of a new eosuchian reptile from the Lower Jurassic of South Wales. *Zool J Linn Soc* 1990, 70:203-264.**

Evans SE: **The postcranial skeleton of the Lower Jurassic eosuchian *Gephyrosaurus bridensis*.** *Zool J Linn Soc* 1981a, **73:**81-116.

Evans SE: **Caudal autotomy in a Lower Jurassic eosuchian.** 1981b, **1981:**883-884.

Evans SE: **Mandibular fracture and inferred behavior in a fossil reptile.** *Copeia* 1983, **1983:** 845-847.

Evans SE: **The classification of the Lepidosauria.** *Zool J Linn Soc* 1984, **82:**87-100.

Evans SE: **Tooth replacement in the Lower Jurassic lepidosaur *Gephyrosaurus bridensis*.** *Neues Jahrb Geol Paläontol Abh* 1985, **1985:**411-420.

Evans SE: **The early history and relationships of the Diapsida.** In *The Phylogeny and Classification of the Tetrapods*. Edited by Benton MJ. Oxford: Oxford University Press; 1988:221-253.

Evans SE: **A new lizard−like reptile (Diapsida: Lepidosauromorpha) from the Middle Jurassic of Oxfordshire.** *Zool J Linn Soc* 1991, **103:**391-412.

Evans SE: **At the feet of the dinosaurs: The early history and radiation of lizards.** *Biol Rev* 2003 **78:**513-551.

Evans SE: **The skull of lizards and tuatara.** In *Biology of the Reptilia, Vol.20, Morphology H: the skull of Lepidosauria*. Edited by Gans C, Gaunt A S, Adler K. Ithaca, New York: Society for the Study of Amphibians and Reptiles; 2008:1-344.

**Evans SE: An early kuehneosaurid reptile (Reptilia: Diapsida) from the Early Triassic of Poland. *Palaeontol Pol* 2009, 65:145-178.**

**Evans** **SE,** Borsuk-Białynicka M: **A small lepidosauromorph reptile from the Early Triassic of Poland.** Palaeontol Pol 2009, **65:**179-202.

Evans SE, Chure DJ: **Upper Jurassic lizards from the Morrison Formation of Dinosaur National Monument.** In Gillete DD ed. Vertebrate Paleontology in Utah: Miscellaneous Publication 99-1 Utah Geological Survey, p. 151-159.

Evans SE, Jones MEH, Matsumoto R: **A new lizard skull from the Purbeck Limestone Formation (Lower Cretaceous) of England.** *Bulletin de la Société Géologique de France* 2012, **183:**517-524.

Evans SE, King MS: **A new specimen of *Protorosaurus* (Reptilia: Diapsida) from the Marl Slate (late Permian) of Britain.** *Yorkshire Geol Soc* 1993, **49:**229-234.

Evans SE, Wang Y: **The Early Cretaceous lizard *Dalinghosaurus* from China.** *Acta Pal Pol* 2005 **50:**725-742.

Evans SE, Prasad GVR, Manhas B: **Rhynchocephalians (Reptilia : Lepidosauria) from the Mesozoic Kota Formation of India.** *Zool J Linn Soc* 2001, **133:**309-334.

Fejfar O, Schleich HH: **Ein Chamäleonfund aus dem unteren Orleanium des Braunkohlen-Tagebaus Merkur-Nord (Nordböhmen).** *Courier Forschungsinstitut Senckenberg* 1994, **173:**167-173.

Fraser NC: **A new rhynchocephalian from the British Upper Trias.** *Palaeontol* 1982, **25:**709-725.

Fraser NC: **New Triassic sphenodontids from South West England and a review of their classification.** *Palaeontol* 1986, **29:**165-186.

Fraser NC: **The osteology and relationships of *Clevosaurus* (Reptilia: Sphenodontida).** *Phil Trans R Soc B* 1988, **321:**125-178.

Fraser NC, Benton MJ: **The Triassic reptiles *Brachyrhinodon* and *Polysphenodon* and the relationships of the sphenodontids.** *Zool J Linn Soc* 1989, **96:**413-445

Fraser NC, Walkden GM: **The postcranial skeleton of the Upper Triassic sphenodontid *Planocephalosaurus robinsonae*.** *Palaeontology* 1984, **27:**575-595.

Gale AS, Huggett JM, Laurie PE, Hailwood EA, Hardenbol J: **Correlation of Eocene–Oligocene marine and continental records: orbital cyclicity, magnetostratigraphy and sequence stratigraphy of the Solent Group, Isle of Wight, UK.** *J Geol Soc* 2006, **163:**401-415.

Gao K, Norell MA: **Taxonomic composition and systematics of the Late Cretaceous lizard assemblages from Ukhaa Tolgod and adjacent localities, Mongolian Gobi Desert.** *Bull Am Mus Nat* 2000, 249:1-118.

García-Veigas J, Cendón DI, Pueyo JJ, Peryt TM: **Zechstein saline brines in Poland, evidence of overturned anoxic ocean during the Late Permian mass extinction event.** *Chem Geol* 2011, **290:**189-201.

Gauthier JA: **Fossil xenosaurid and anguid lizards from the early Eocene Wasatch Formation, Southeast Wyoming, and a revision of the Anguioidea.** *Rocky Mountain Geol* 1982, **21:**7-54

Gauthier JA, Estes R, deQueiroz K: **A phylogenetic analysis of the Lepidosauromorpha.** In *Phylogenetic relationships of the lizard families: essays commemorating Charles L. Camp*. Edited by Estes R, Pregill G. Stanford, CA, USA: Stanford University Press; 1988:15-98.

Gorr TA, Mable BK, Kelinschmidt T: **Phylogenetic analysis of reptilian hemoglobins: tree, rates, and divergences.** J Mol Evol 1998, **47:**471-485.

Gottmann-Quesada A, Sander M: **A redescription of the early archosauromorph *Protorosaurus speneri* MEYER 1932.** *Palaeontographica Abt. A* 2009, **287:**123-220.

Gow CE: **The morphology and relationships of *Youngina capensis* Broom and *Prolacerta broomi* Parrington.** *Palaeont Afr* 1975, **18:**89-131.

Gradstein FM, Ogg JG: **Geologic Time Scale 2004 – why how and where next!** *Lethaia* 2004, **37:**175-181.

Gradstein FM, Ogg JG, Smith AG, Agterberg FP, Bleeker W, Cooper RA, Davydov V, Gibbard P, Hinnov LA, House MR, Lourens LJ, Luterbacher H-P, McArthur J, Melchin MJ, Robb LJ, Shergold J, Villeneuve M, Wardlaw BR, Ali J, Brinkhuis H, Hilgen FJ, Hooker J, Howarth RJ, Knoll AH, Laskar J, Monechi S, Powell J, Plumb KA, Raffi I, Röhl U, Sanfilippo A, Schmitz B, Shackleton NJ, Shields GA, Strauss H, Van Dam J, Veizer J. van Kolfschoten TH, Wilson D. *Geologic Time Scale*. Cambridge: Cambridge University Press; 2004.

Gradstein FM, Ogg JG, Schmitz M, Ogg G: *The geological time scale 2012*. Amsterdam, Elsevier; 2012.

Grande L, Bucheim HP. **Paleontological and sedimentological variation in Early Eocene fossil lake.** *Contr Geol Uni Wyoming* 1994, **30:**33-56.

Günther A: **Contribution to the anatomy of *Hatteria* (*Rhynchocephalus*, Owen).** *Phil Trans R Soc Lond* **157:**1-34.

Hecht M: A new xantusiid lizard from the Eocene of Wyoming. *Amer Mus Novit* 1956, **1774:**1-8.

Heckert AB, Lucas SG, Rinehart LF, Hunt AP: **A new genus and species of sphenodontian from the Ghost Ranch *Coelophysis* Quarry (Upper Triassic: Apachean), Rock Point Formation, New Mexico, USA.** *Palaeontol* 2008 **51:**827-845.

Hill RV: **Integration of morphological data sets for phylogenetic analysis of Amniota: the importance of integumentary characters and increased taxonomic sampling.** *Syst Biol* 2005, **54:**530-547.

Hipsley CA, Himmelmann L, Metzler D, Müller J: **Integration of Bayesian molecular clock methods and fossil-based soft bounds reveals early Cenozoic origin of African lacertid lizards.** *BMC Evol Biol* 2009, **9,151:**1-13.

Hooker JJ, Grimes ST, Mattey DP, Collinson ME, Sheldon ND. **Refined correlation of the UK Late Eocene-Early Oligocene Solent Group and timing of its climate history.** *Geol Soc Am Sp Pap* 2009, **452:**179-195.

Huber P, Lucas SG, Hunt AP: **Vertebrate biochronology of the Newark Supergroup, Triassic, eastern NorthAmerica.** In *The Nonmarine Triassic*. Edited by Lucas SG, Morales M. *New Mex Mus Nat Hist Sci Bull* 1993 **3:**179-186.

Hugall AF, Foster R, Lee MS: **Calibration choice, rate smoothing, and the pattern of tetrapod diversification according to the long nuclear gene RAG-1.** Syst Biol 2007, **56:**543-563.

Janke A, Erpenbeck D, Nilsson M, Arnason U: **The mitochondrial genomes of the iguana (*Iguana iguana*) and the caiman (*Caiman crocodylus*): implications for amniote phylogeny.** *Proc R Soc Lond B* 2001, **268:**623-631.

Jones MEH: **The evolution of skull shape and feeding strategy in Rhynchocephalia (Diapsida: Lepidosauria).** J Morphol. 2008, **269:**945-966.

Jones MEH, Tennyson AJD, Worthy JP, Evans SE, Worthy TH: **A sphenodontine (Rhynchocephalia) from the Miocene of New Zealand and palaeobiogeography of the tuatara (*Sphenodon*).** *Proc Roy Soc B* 2009, **276:**1385–1390.

Kearney M: **Systematics of the Amphisbaenia (Lepidosauria: Squamata) based on morphological evidence from recent and fossil forms.** *Herp Mono* 2003, **17:**1-74

Klembara J: **Beitrag zur Kenntnis der Subfamilie Anguinae (Reptilia, Anguidae).** *Acta Universitis Carolinae, Geologica* 1981, **2:**121-168.

Klembara J, Green B: **Anguimorph lizards (Squamata, Anguimorpha) from the Middle and Late Eocene of the Hampshire Basin of southern England.** *J Syst Palaeontol* 2010, **8:**97-129.

Kowalis BJ, Christiansen EH, Deino AL, Peterson F, Turner CE, Kunk MJ, Obradovich JD: **The age of the Morrison Formation.** *Mod Geol* 1998, **22:**235-260.

Kozur HW, Bachmann GH: **Correlation of the Germanic Triassic with the international scale.** *Albertiana* 2005, **32:**21-35.

Kozur HW, Bachman GH: **Updated correlation of the Germanic Triassic with the Tethyan scale and assigned numeric ages**. *Berichte* *Geol* 2008, **76**:53-58.

Krassilov V, Karasev E: **Paleofloristic evidence of climate change near and beyond the Permian-Triassic boundary.** *Palaeogeog Palaeoclim Palaeoecol* 2009, **284:**326-336.

Kumar S, Hedges SB: **A molecular timescale for vertebrate evolution.** *Nature* 1998, 917–920.

Kumazawa Y: **Mitochondrial genomes from major lizard families suggest their phylogenetic relationships and ancient radiations.** *Gene* 2007, 388: 19–26.

Kumazawa Y, Nishida N: **Complete mitochondrial DNA sequences of the green turtle and blue-tailed mole skink: statistical evidence for archosaurian affinity of turtles.** *Mol Biol Evol* 1999, **16:**784-792.

Lee MSY: **The origin of the turtle body plan: bridging a famous morphological gap.** *Science* 1992, 261: 1716-1720.

Lee MSY: **Pareiasaur phylogeny and the origin of turtles.** *Zool J Linn Soc* 1997, **120:**197-280.

Lee MSY: **Molecules, morphology, and the monophyly of diapsid reptiles.** *Contrib Zool* 2001, **70:**1.

Lofgren DL, Lillegraven JA, Clemens WA, Gingerich PD, Williamson TE: **Paleocene biochronology of North America: the Puercan through Clarkforkian land mammal ages.** In *Late Cretaceous and Cenozoic mammals of North America: biostratigraphy and geochronology*. Edited by Woodburne MO. New York: Columbia University Press; 2004:43-105.

Lofgren DL, Scherer BE, Clark CK, Standhardt B: **First Record of *Stygimys* (Mammalia, Multituberculata, Eucosmodontidae) from the Paleocene (Puercan) part of the North Horn Formation, Utah, and a review of the genus.** *J Mamm Evol* 2005, **12:**77-97.

Longrich NR, Bhullar B-AS, Gauthier JA: **A transitional snake from the Late Cretaceous period of North America.** *Nature* 2013, **488:**205-208.

Lyson TR, Bever GS, Bhullar BS, Joyce WG, Gauthier JA: **Transitional fossils and the origin of turtles.** *Biol Lett* 2010, **6:**830-833.

Lyson TR, Sperling EA, Heimberg AM, Gauthier JA, King BL, Peterson KJ: **MicroRNAs support a turtle + lizard clade.** *Biol Lett* 2011, **8:**104-107.

Magallón SA: **Dating lineages: molecular and paleontological approaches to the temporal framework of clades.** *Int J Plant Sci* 2004, **165S:**7-21.

Menning, M., Gast, R., Hagdorn, H., Käding, K.K, Simon, T., Szurlies, M. and Nitsch, E: **Zeitskala für Perm und Trias in der Stratigraphischen Tabelle von Deutschland 2002, zyklostratigraphische Kalibrierung der höheren Dyas und Germanischen Trias und das Alter der Stufen Roadium bis Rhaetium 2005.** *News Strat* 2005, **41:**173-210.

Modesto SP, Sues HD: **The skull of the Early Triassic archosauromorph reptile *Prolacerta broomi* and its phylogenetic significance.** *Zool J Linn Soc 2004*, **140**:335-351.

Moody S, Rocek Z: ***Chamaeleo caroliquarti* (Chamaeleonidae, Sauria): a new species from the Lower Miocene of central Europe.** *Vestnik Ustfedniho ustavu geologickeho* 1980, **55:**85-92

Müller J: **Early loss and multiple return of the lower temporal arcade in diapsid reptiles.** *Naturwissen* 2003, **90:**473-476.

Müller J: **The relationships among diapsid reptiles and the influence of taxon selection.** – In *Recent Advances in the Origin and Early Radiation of Vertebrates*. Edited by Arratia, G, Cloutier R. Wilson VH. München: Dr. Friedrich Pfeil; 2004:379-408,

Müller J, Reisz RR: **Four well-constrained calibration points from the vertebrate fossil record for molecular clock estimates.** *BioEssays* 2005, **27:**1069-1075.

Mundil R, Pálfy J, Renne PR, Brack P: **The Triassic timescale: new constraints and a review of geochronological data.** *Geol Soc London Spec Publ* **334:**41-60.

Nesbitt SJ: **The osteology of the Middle Triassic pseudosuchian archosaur *Arizonasaurus babbitti*.** Hist Biol 2005, **17:**19-47.

Nesbitt SJ: **The early evolution of archosaurs: relationships and the origin of major clades.** *Bull Am Mus Nat Hist* 2011, **352:**1-292.

Nessov LA: **Rare bony fishes, terrestrial lizards and mammals from the lagoonal zone of the littoral lowlands of the Cretaceous of the Kyzylkumy.** *Yearbook All-Union Palaeont Soc, Leningrad* 1985, **28:**199-219

Nydam RL: **A new taxon of helodermatid-like lizard from the Albian-Cenomanian of Utah.** *J Vertebr Paleontol* 2000, **20:**285-294.

Ogg JG, Smith AG: **The geomagnetic polarity time scale.** In *A geological time scale*. Edited by Gradstein FM, Ogg JG, Smith AG. Cambridge, UK: Cambridge University Press; 2004:63-86.

Okajima Y, Kumazawa Y: **Mitogenomic perspectives into iguanid phylogeny and biogeography: Gondwanan vicariance for the origin of Madagascan oplurines.** *Gene* 2009, **441:**28-35.

Olsen PE, McCune AR, Thomson KS: **Correlation of the early Mesozoic Newark Supergroup by vertebrates, principally fishes.** *Am J Sci* 1982, **82:**1-44.

Parham JF, Irmis RB: **Caveats on the use of fossil calibrations for molecular dating: a comment on Near et al.** 2008, **171:**132-136.

Parham JF, Donoghue PCJ, Bell CJ, Calway TD, Head JJ, Holroyd PA, Inoue JG, Irmis RB, Joyce WG, Ksepka DT, Patané JSL, Smith ND, Tarver JE, van Tuinen M, Yang Z, Angielczyk KD, Greenwood JM, Hipsley CA, Jacobs L, Makovicky PJ, Müller J, Smith KT, Theodor JM, Warnock RCM, Benton MJ: **Best practices for justifying fossil calibrations.** *Syst Biol* 2013, **61:**346-359.

Peppe DJ, Evans DAD, Smirnov AV: **Magnetostratigraphy of the Ludlow Member of the Fort Union Formation (Lower Paleocene) in the Williston Basin, North Dakota.** *Geol Soc Am Bull* 2009, **121:**65-79.

Pettigrew TH: **A gliding reptile from the Upper Permian of North East England.** *Nature* 1979, **281:**297-298

Prothero DR, Estes R: **Late Jurassic lizards from Como Bluff Wyoming and their paleobiogeographic significance.** *Nature* 1981, **286:**484-486.

Reisz RR: ***Petrolacosaurus*, the oldest known diapsid reptile.** Science 1977, **196:**1091-1093.

Reisz RR, Müller J: **Molecular timescales and the fossil record: a paleontological perspective.** *TRENDS Gen* 2004, **20:** 237-241.

Reisz RR, Mueller J, Tsuji L, Scott D: **The cranial osteology of *Belebey vegrandis* (Parareptilia: Bolosauridae), from the Middle Permian of Russia, and its bearing on reptilian evolution.** *Zool J Linn Soc* 2007, **151:**191-214.

Reisz RR, Laurin M, Marjanović D: ***Apsisaurus witteri* from the Lower Permian of Texas: yet another small varanopid synapsid, not a diapsid.** *Journal of Vertebrate Paleontology* 2010, **30**:1628-1631.

Rest JS, Ast JC, Austin CC, Waddell PJ, Tibbetts EA, Hay JM, Mindell DP: **Molecular systematics of primary reptilian lineages and the tuatara mitochondrial genome.** *Mol Phylogenet* 2003, **29**:289-297.

Reynoso VH: A **Middle Jurassic *Sphenodon*-like sphenodontian (Diapsida: Lepidosauria) from Huizachal Canyon, Tamaulipas, Mexico.** J Vert Paleontol 1996, **16:**210-221.

Reynoso VH: **A “beaded” sphenodontian (Diapsida: Lepidosauria) from the Early Cretaceous of central Mexico.** *J Vertebr Paleontol* 1997, **17:**52-59.

Reynoso VH: **An unusual aquatic sphenodontian (Reptilia: Diapsida) from the Tlayua Formation (Albian), central Mexico.** *J Paleontol* 2000, **74:**133-148.

Reynoso VH: **Possible evidence of a venom apparatus in a middle Jurassic sphenodontian from the Huizachal red beds of Tamaulipas, México.** *J Vertebr Paleontol* 2005, **25:**646-654.

Reynoso VH, Clark JM: **A dwarf sphenodontian from the Jurassic la Boca Formation of Tamaulipas, Mexico.** *J Vertebr Paleontol* 1998, **18:**333-339.

Rieppel O: **Lepidosauromorpha: an overview with special emphasis on
the Squamata.** In *In the shadow of the dinosaurs: Early Mesozoic tetrapods*. Edited by Fraser NC, Sues H-D. Cambridge: Cambridge University Press; 1994:23-37.

Rieppel O, Grande L: **The anatomy of the fossil varanid lizard *Saniwa ensidens* Leidy, 1870, based on a newly discovered complete skeleton.** *J Paleontol* 2007, **81:**643-665.

Ritzkowski S: **K-Ar Altersbestimmungen der bernsteinführenden Sedimente des Samlandes (Palaögen, Bezirk Kaliningrad).** In *Metalla, Sonderheft 66 zum Symposium Neue Erkenntnisse zum Bernstein*. Edited by Ganzelewski M, Rehren TH, Slotta R. Bochum, Germany: Deutsches Bergbau Museum; 1997:19-24.

**Robinson PL: Gliding lizards from the Upper Keuper of Great Britain. *Proc Geol Soc* 1962, 1601:137-146.**

Robinson PL**: Triassic vertebrates from lowland and upland.** Sci Cult 1967, **33:**169-173.

Roscher M, Schneider JW: **Permo-Carboniferous climate: Early Pennsylvanian to Late Permian climate development of central Europe in a regional and global context.** *Geological Society of London Special Publications* 2006, **265:**95-136.

Rossman T: **Osteologische Beschreibung von *Geiseltaliellus longicaudus* Kuhn, 1944 (Squamata: Iguanoidea) aus dem Mittleren Eozän der Fossillagerstätten Geiseltal und Grube Messel (Deutschland), mit einer Revision der Gattung *Geiseltaliellus*.** *Palaeontographica Abteilung a Palaeozoologie Stratigraphie*, 2000, **258:**117-158.

Rossmann T: **Messelosaurine lacertilians (Squamata: Iguanoides) from the Palaeogene of France and North America.** *Neues Jahrb Geol Paläontol Monat* 1999, **1999:**577-592.

San Mauro: **A multilocus timescale for the origin of extant amphibians.** Mol Phy Evol 2010, **56:**554-561.

Sanders KL, Lee MSY: **Evaluating molecular clock calibrations using Bayesian analyses with soft and hard bounds.** *Biol Lett* 2007, **3:**275-279.

Schatzinger RA: **New species of “*Palaeoxantusia*” (Reptilia: Sauria) from the Uintan (Eocene) of San Diego Co., Cal.** *J Paleontol* 1980, **54**:460-471.

Schoch RR: **Stratigraphie und Taphonomie wirbeltierreicher Schichten im Unterkeuper (Mitteltrias) von Vellberg (SW-Deutschland).** *Stuttgarter Beitr Naturkd B* 2002, **318:**1-30.

Seiffert J: **Upper Jurassic lizards from central Portugal.** *Memorias Serviços Geológicos de Portugal* 1973, **22:**1-85.

Sennikov AG, Golubev VK: **Vyazniki biotic assemblage of the terminal Permian.** *Paleontolog J* 2006, **40:**475-481.

Shen X-X, Liang D, Wen J-Z, Zhang P: **Multiple genome alignments facilitate development of NPCL markers: a case study of tetrapod phylogeny focusing on the position of turtles.** *Mol Biol Evol* 2011, **28:** 3237-3252.

Smith KT: **Eocene lizards of the clade *Geiseltaliellus* from Messel and Geiseltal, Germany, and the Early radiation of Iguanidae (Reptilia: Squamata).** *Bull Peabody Mus Nat Hist* 2009a, **50:**219-306.

Smith KT: **A new assemblage of squamates from the earliest Eocene (zone W_A_0) of the Bighorn Basin, Wyoming: Biogeography during the warmest interval of the Cenozoic.** *J Syst Palaeontol* 2009b, **7:**299-358.

Smith ME, Carroll AR, Singer BS: **Synoptic reconstruction of a major ancient lake system: Eocene Green River Formation, Western United States.** *GSA Bull* 2008, **120:**54-84.

Smith ME, Chamberlain KR, Singer BS, Carroll AR: **Eocene clocks agree: coeval 40Ar/ 39Ar, U-Pb, and astronomical ages from the Green River Formation.** *Geol* 2010, **38:**527-530.

Steininger FF, Wessely G: **From the Tethyan Ocean to the Paratethys Sea: Oligocene to Neogene stratigraphy, paleogeography and paleobiogeography of the circum-Mediterranean region and the Oligocene to Neogene Basin evolution in Austria.** *Mitt Osterr Geol Ges* 2000, **92:**95-116.

Sues H-D, Hopson JA: **Anatomy and phylogenetic relationships of *Boreogomphodon jeffersoni* (Cynodontia: Gomphodontia) from the Upper Triassic of Virginia.** *J Vertebr Paleontol* 2010, **30:**1202-1220.

Sues H-D, Munk W: **A remarkable assemblage of terrestrial tetrapods from the Zechstein (Upper Permian: Tatarian) near Korbach (northwestern Hesse).** Palaeontologische Zeitschrift 1996, **70(1/2):**213-223.

**Sues H-D, Olsen PE: Triassic vertebrates of Gondwanan aspect from the Richmond Basin of Virginia. *Science* 1990, 249:1020-**1023.

Sues H-D, Shubin NH, Olsen PE: **A new sphenodontian (Lepidosauria: Rhynchocephalia) from the McCoy Brook Formation (Lower Jurassic) of Nova Scotia. Canada.** *J Vert Paleontol* 1994, **14:**327-340.

Sullivan RM: **Lizards from Swain Quarry "Fort Union Formation," Middle Paleocene (Torrejonian), Carbon County, Wyoming.** *J Paleontol* 1982, **56:**996-1010.

Sullivan RM: **A New Middle Paleocene (Torrejonian) rhineurid amphisbaenian, *Plesiorhineura tsentasi* new genus, new species, from the San Juan Basin, New Mexico.** *J Palaeontol* 1985, 59:1481-1485.

Sullivan RM: **Caudata and Squamata from Gidley and Silberling Quarries, Montana.** *J Vertebr Paleontol* 1991, **11:**293-301.

Sullivan R, Keller T, Habersetzer J: **Middle Eocene (Geiseltalian) anguid lizards from Geiseltal and Messel, Germany. I. *Ophisauriscus quadrupes* Kuhn 1940.** *Courier Forschungsinstitut Senckenberg* 1999, **216:**97-129.

Tatarinov LP: **Discovery of pseudosuchians in the Upper Permian of the USSR.** *Paleontologicheskii Zhurnal* 1960, **1960:**74-80. [in Russian]

Townsend TM, Larson A, Louis E, Macey JR: **Molecular phylogenetics of Squamata: the position of snakes, amphisbaenians, and dibamids, and the root of the squamate tree.** *Syst Biol* 2004, **53:**735-757.

Travouillon KJ, Legendre S, Archer M, Hand SJ: **Palaeoecological analyses of Riversleigh's Oligo-Miocene sites: implications for and Oligo-Miocene climate change in Australia.** *Palaeogeog Palaeoclim Palaeoecol* 2009, **276:**24-37

Tremaine K: **Examining the Torrejonian (To2-To3) boundary in the Nacimiento Formation, San Juan Basin, New Mexico.** *Geol Soc Am Abstr Prog* 2011, **43:**120.

Vaughan DJ, Sweeney MA, Friedrich G, Diedel R, Haranczyk C: **The Kupferschiefer; an overview with an appraisal of the different types of mineralization.** *Econ Geol* 1989, **84:**1003-1027.

Vidal N, Hedges SB: **The phylogeny of squamate reptiles (lizards, snakes, and amphisbaenians) inferred from nine nuclear protein-coding genes.** *C R Biol* 2005, **328:**1000-1008.

Waldman M, Evans SE: **Lepidosauromorph reptiles from the Middle Jurassic of Skye.** *Zool J Linn Soc* 1994, **112:**135-150.

Wang, X.L. and Zhou, Z.H: **Mesozoic Pompei.** In *The Jehol Biota*. Edited by Chang MM, Chen PJ, Wang YQ, Wang Y. Shanghai: Shanghai Scientific and Technical Publishers; 2003:19-35.

Werneburg I, Sánchez-Villagra MR: **Timing of organogenesis support basal position of turtles in the amniote tree of life.** BMC Evol Biol 2009, **9:**82

Whiteside DI: **The head skeleton of the Rhaetian sphenodontid *Diphydontosaurus avonis* gen. et sp. nov., and the modernising of a living fossil.** *Phil. Trans R Soc B* 1986, **312:**379-430.

Wiens JJ, Brandley MC, Reeder TW: **Why does a trait evolve multiple times within a clade? Repeated evolution of snakelike body form in squamate reptiles.** *Evolution* 2006, 60: 123–141.

Wiens JJ, Kuczynski CA, Townsend T, Reeder TW, Mulcahy DG, Sites Jr. JW: **Combining phylogenomics and fossils in higher-level squamate reptile phylogeny: molecular data change the placement of fossil taxa.** *Syst Biol* 2010, **59:**1-15.

Williamson TE, Nichols DJ, Weil A: **Paleocene palynomorph assemblages from the Nacimiento Formation, San Juan Basin, New Mexico, and their biostratigraphic significance**. *New Mex Geol* 2008, **30:**3-11.

Wolfe AP, Tappert R, Muehlenbachs K, Boudreau M, McKellar RC, Basinger JF, Garrett A: **A new proposal concerning the botanical origin of Baltic amber.** *Proc R Soc B* 2009, **276:**3403-3412.

Woodburne MO, MacFadden BJ, Case JA, Springer MS, Pledge NS, Power JD, Woodburne JM, Springer KB: **Land mammal biostratigraphy and magnetostratigraphy of the Etadunna Formation (Late Oligocene) of South Australia.** *J Vertebr Paleontol* 1993, **134:**483–515.

Wu X-C: **The discovery of a new thecodont from north east Shanxi.** *Vertebr PalAsiatica* 1981, **19:**122-132.

Wu X-C: **Late Triassic-Early Jurassic sphenodontians from China and the phylogeny of the Sphenodontia.** In *In the shadow of the dinosaurs: Early Mesozoic tetrapods*. Edited by Fraser NC, Sues H-D. Cambridge: Cambridge University Press; 1994:38–69.

Zardoya R, Meyer A: **Complete mitochondrial genome suggests diapsid affinities of turtles.** *Proc Nat Acad Sci* 1998, **95:**4226-14231.

Zardoya R, Meyer A: **The evolutionary position of turtles revisited.** *Naturwissenschaften* 2001, **88:**193-200.
