# Supplementary material for: Integration of molecules and new fossils supports a Triassic origin for Lepidosauria (lizards, snakes, and tuatara)
Source: BMC Evol Biol. 2013 Sep 25;13:208. doi: 10.1186/1471-2148-13-208 (PMC4016551; doi:10.1186/1471-2148-13-208)
Supplement: Additional file 4: — List of sequences used from GenBank for 77 extant amniote taxa. [file 1471-2148-13-208-S4.docx]

Text S2 List of sequences used from GenBank

We used RAG1 sequences from 77 extant taxa (Rhynchocephalia: 1; Squamata: 62; Testudines: 4; Aves: 4; Crocodylia: 3; Mammalia 2):

| Taxon | RAG 1 |
| --- | --- |
| *Acontias meleagris* | [AY662639](http://www.ncbi.nlm.nih.gov/nuccore/AY662639) |
| *Alligator mississipiensis* | [AF143724](http://www.ncbi.nlm.nih.gov/nuccore/AF143724) |
| *Amphisbaena xera* | [AY662619](http://www.ncbi.nlm.nih.gov/nuccore/AY662619) |
| *Anniella pulchra* | [AY662605](http://www.ncbi.nlm.nih.gov/nuccore/AY662605) |
| *Anolis paternus* | [AY662589](http://www.ncbi.nlm.nih.gov/nuccore/AY662589) |
| *Aspidoscelis tigris* | [AY662620](http://www.ncbi.nlm.nih.gov/nuccore/AY662620) |
| *Asymblepharus sikimmensis* | [AY662631](http://www.ncbi.nlm.nih.gov/nuccore/AY662631) |
| *Basiliscus plumifrons* | [AY662599](http://www.ncbi.nlm.nih.gov/nuccore/AY662599) |
| *Bipes biporus* | [AY662616](http://www.ncbi.nlm.nih.gov/nuccore/AY662616) |
| *Brookesia thieli* | [AY662577](http://www.ncbi.nlm.nih.gov/nuccore/AY662577) |
| *Calotes calotes* | [AY662584](http://www.ncbi.nlm.nih.gov/nuccore/AY662584) |
| *Calumma brevicornis* | [AY662579](http://www.ncbi.nlm.nih.gov/nuccore/AY662579) |
| *Carettochelys insculpta* | [AY687904](http://www.ncbi.nlm.nih.gov/nuccore/AY687904) |
| *Celestus enneagrammus* | [AY662604](http://www.ncbi.nlm.nih.gov/nuccore/AY662604) |
| *Chalarodon madagascariensis* | [FJ356745](http://www.ncbi.nlm.nih.gov/nuccore/FJ356745) |
| *Chalcides ocellatus* | [AY662638](http://www.ncbi.nlm.nih.gov/nuccore/AY662638) |
| *Chamaeleo rudis* | [AY662578](http://www.ncbi.nlm.nih.gov/nuccore/AY662578) |
| *Chelonia mydas* | [AY687907](http://www.ncbi.nlm.nih.gov/nuccore/AY687907) |
| *Cordylus polyzonus* | [AY662643](http://www.ncbi.nlm.nih.gov/nuccore/AY662643) |
| *Crenadactylus ocellatus* | [AY662627](http://www.ncbi.nlm.nih.gov/nuccore/AY662627) |
| *Crocodylus cataphractus* | [AY239174](http://www.ncbi.nlm.nih.gov/nuccore/AY239174) |
| *Ctenophorus salinarum* | [AY662580](http://www.ncbi.nlm.nih.gov/nuccore/AY662580) |
| *Ctenotus robustus* | [AY662630](http://www.ncbi.nlm.nih.gov/nuccore/AY662630) |
| *Cylindrophis ruffus* | [AY662613](http://www.ncbi.nlm.nih.gov/nuccore/AY662613) |
| *Dibamus sp.* | [AY662645](http://www.ncbi.nlm.nih.gov/nuccore/AY662645) |
| *Dinodon sp* | [AY662611](http://www.ncbi.nlm.nih.gov/nuccore/AY662611) |
| *Elgaria panamintina* | [AY662603](http://www.ncbi.nlm.nih.gov/nuccore/AY662603) |
| *Enyalioides laticeps* | [AY662593](http://www.ncbi.nlm.nih.gov/nuccore/AY662593) |
| *Eremias sp.* | [AY662615](http://www.ncbi.nlm.nih.gov/nuccore/AY662615) |
| *Eublepharis turcmenicus* | [AY662622](http://www.ncbi.nlm.nih.gov/nuccore/AY662622) |
| *Eumeces anthracinus* | [AY662634](http://www.ncbi.nlm.nih.gov/nuccore/AY662634) |
| *Euprepis auratus* | [AY662629](http://www.ncbi.nlm.nih.gov/nuccore/AY662629) |
| *Feylinia polylepis* | [AY662637](http://www.ncbi.nlm.nih.gov/nuccore/AY662637) |
| *Gallus gallus* | [M58530](http://www.ncbi.nlm.nih.gov/nuccore/M58530) |
| *Gambelia wislizenii* | [AY662600](http://www.ncbi.nlm.nih.gov/nuccore/AY662600) |
| *Gavialis gangeticus* | [AF143725](http://www.ncbi.nlm.nih.gov/nuccore/AF143725) |
| *Gekko gecko* | [AY662625](http://www.ncbi.nlm.nih.gov/nuccore/AY662625) |
| *Geochelone pardalis* | [AY687912](http://www.ncbi.nlm.nih.gov/nuccore/AY687912) |
| *Gloydius halys* | [AY662614](http://www.ncbi.nlm.nih.gov/nuccore/AY662614) |
| *Heloderma suspectum* | [AY662606](http://www.ncbi.nlm.nih.gov/nuccore/AY662606) |
| *Hoplocercus spinosus* | [AY662592](http://www.ncbi.nlm.nih.gov/nuccore/AY662592) |
| *Hydrosaurus sp.* | [AY662583](http://www.ncbi.nlm.nih.gov/nuccore/AY662583) |
| *Japalura tricarinata* | [AY662585](http://www.ncbi.nlm.nih.gov/nuccore/AY662585) |
| *Lanthanotus borneensis* | [AY662609](http://www.ncbi.nlm.nih.gov/nuccore/AY662609) |
| *Leiocephalus carinatus* | [AY662598](http://www.ncbi.nlm.nih.gov/nuccore/AY662598) |
| *Leiolepis belliana* | [AY662587](http://www.ncbi.nlm.nih.gov/nuccore/AY662587) |
| *Leposoma parietale* | [AY662621](http://www.ncbi.nlm.nih.gov/nuccore/AY662621) |
| *Lialis jicari* | [AY662628](http://www.ncbi.nlm.nih.gov/nuccore/AY662628) |
| *Ophisaurus attenuatus* | [AY662602](http://www.ncbi.nlm.nih.gov/nuccore/AY662602) |
| *Oplurus cuvieri* | [AY662601](http://www.ncbi.nlm.nih.gov/nuccore/AY662601) |
| *Ornithorhynchus anatinus* | [EF551559](http://www.ncbi.nlm.nih.gov/nuccore/EF551559) |
| *Passer montanus* | [AF143738](http://www.ncbi.nlm.nih.gov/nuccore/AF143738) |
| *Phrynocephalus raddei* | [AY662586](http://www.ncbi.nlm.nih.gov/nuccore/AY662586) |
| *Phrynosoma mcallii* | [AY662590](http://www.ncbi.nlm.nih.gov/nuccore/AY662590) |
| *Physignathus cocincinus* | [AY662582](http://www.ncbi.nlm.nih.gov/nuccore/AY662582) |
| *Platysternon megacephalum* | [AY687905](http://www.ncbi.nlm.nih.gov/nuccore/AY687905) |
| *Proscelotes eggeli* | [AY662636](http://www.ncbi.nlm.nih.gov/nuccore/AY662636) |
| *Pseudothecadactylus lindneri* | [AY662626](http://www.ncbi.nlm.nih.gov/nuccore/AY662626) |
| *Python reticulatus* | [EU624119](http://www.ncbi.nlm.nih.gov/nuccore/EU624119) |
| *Ramphotyphlops braminus* | [AY662612](http://www.ncbi.nlm.nih.gov/nuccore/AY662612) |
| *Rhineura floridana* | [AY662618](http://www.ncbi.nlm.nih.gov/nuccore/AY662618) |
| *Sauromalus ater* | [AY662591](http://www.ncbi.nlm.nih.gov/nuccore/AY662591) |
| *Scelotes anguina* | [AY662635](http://www.ncbi.nlm.nih.gov/nuccore/AY662635) |
| *Shinisaurus crocodylurus* | [AY662610](http://www.ncbi.nlm.nih.gov/nuccore/AY662610) |
| *Sphaerodactylus shrevei* | [AY662623](http://www.ncbi.nlm.nih.gov/nuccore/AY662623) |
| *Sphenodon punctatus* | [AY662576](http://www.ncbi.nlm.nih.gov/nuccore/AY662576) |
| *Struthio camelus* | [AF143727](http://www.ncbi.nlm.nih.gov/nuccore/AF143727) |
| *Tachyglossus aculeatus* | [EF551558](http://www.ncbi.nlm.nih.gov/nuccore/EF551558) |
| *Teratoscincus przewalskii* | [AY662624](http://www.ncbi.nlm.nih.gov/nuccore/AY662624) |
| *Tinamus guttatus* | [AF143726](http://www.ncbi.nlm.nih.gov/nuccore/AF143726) |
| *Trogonophis wiegmanni* | [AY662617](http://www.ncbi.nlm.nih.gov/nuccore/AY662617) |
| *Typhlosaurus lomii* | [AY662641](http://www.ncbi.nlm.nih.gov/nuccore/AY662641) |
| *Uromastyx acanthinura* | [AY662588](http://www.ncbi.nlm.nih.gov/nuccore/AY662588) |
| *Varanus griseus* | [AY662608](http://www.ncbi.nlm.nih.gov/nuccore/AY662608) |
| *Xantusia vigilis* | [AY662642](http://www.ncbi.nlm.nih.gov/nuccore/AY662642) |
| *Xenosaurus grandis* | [AY662607](http://www.ncbi.nlm.nih.gov/nuccore/AY662607) |
| *Zonosaurus sp.* | [AY662644](http://www.ncbi.nlm.nih.gov/nuccore/AY662644) |
