# Supplementary figures and images for: Integration of molecules and new fossils supports a Triassic origin for Lepidosauria (lizards, snakes, and tuatara)
Source: BMC Evol Biol. 2013 Sep 25;13:208. doi: 10.1186/1471-2148-13-208 (PMC4016551; doi:10.1186/1471-2148-13-208)

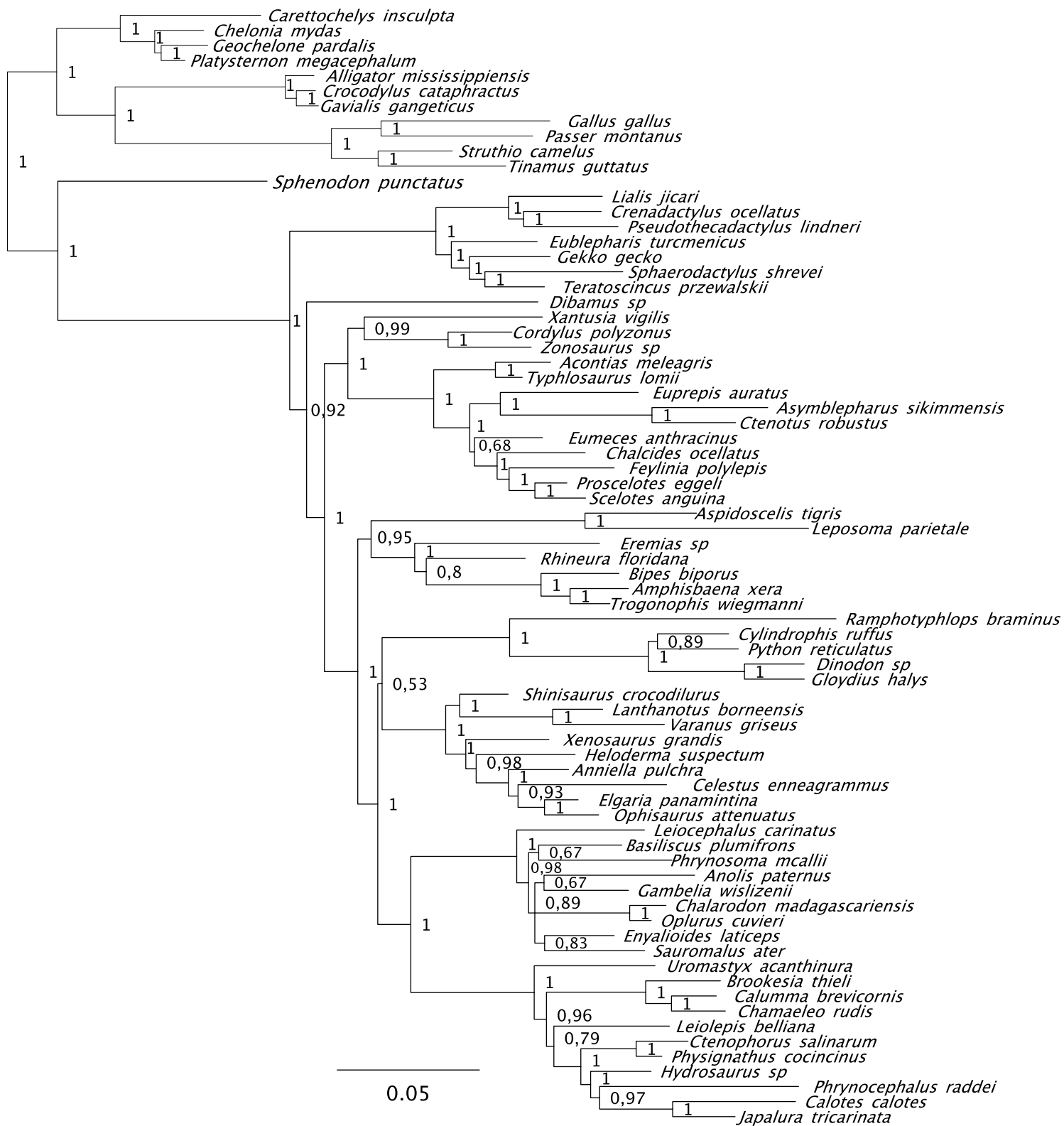

Supplement: Additional file 5: — Majority rule consensus tree from MrBayes, phylogram with posterior probabilities shown. [file 1471-2148-13-208-S5.pdf]
